# Eicosanoids in the Pancreatic Tumor Microenvironment—A Multicellular, Multifaceted Progression

**DOI:** 10.1016/j.gastha.2022.02.007

**Published:** 2022-06-11

**Authors:** Vikas B. Gubbala, Nidhi Jytosana, Vincent Q. Trinh, H. Carlo Maurer, Razia F. Naeem, Nikki K. Lytle, Zhibo Ma, Steven Zhao, Wei Lin, Haiyong Han, Yu Shi, Tony Hunter, Pankaj K. Singh, Kenneth P. Olive, Marcus C.B. Tan, Susan M. Kaech, Geoffrey M. Wahl, Kathleen E. DelGiorno

**Affiliations:** 1 Gene Expression Laboratory, Salk Institute for Biological Studies, La Jolla, California; 2 Department of Cell and Developmental Biology, Vanderbilt University, Nashville, Tennessee; 3 Department of Surgery, Vanderbilt University Medical Center, Nashville, Tennessee; 4 Department of Medicine, Herbert Irving Comprehensive Cancer Center, Columbia University Irving Medical Center, New York, New York; 5 Internal Medicine II, School of Medicine, Technische Universität München, Munich, Germany; 6 Immunobiology and Microbial Pathogenesis Laboratory, Salk Institute for Biological Studies, La Jolla, California; 7 Molecular Medicine Division, Translational Genomics Research Institute, Phoenix, Arizona; 8 Molecular and Cell Biology Laboratory, Salk Institute for Biological Studies, La Jolla, California; 9 Eppley Institute for Research in Cancer, University of Nebraska Medical Center, Omaha, Nebraska; 10 Vanderbilt Digestive Disease Research Center, Vanderbilt University Medical Center, Nashville, Tennessee; 11 Vanderbilt Ingram Cancer Center, Nashville, Tennessee

**Keywords:** Tuft Cells, Prostaglandins, PTGES, PTGIS, TBXAS1

## Abstract

**Background and Aims:**

Eicosanoids, oxidized fatty acids that serve as cell-signaling molecules, have been broadly implicated in tumorigenesis. Here, we aimed to identify eicosanoids associated with pancreatic tumorigenesis and the cell types responsible for their synthesis.

**Methods:**

We profiled normal pancreas and pancreatic ductal adenocarcinoma in mouse models and patient samples using mass spectrometry. We interrogated RNA sequencing data sets for eicosanoid synthase or receptor expression. Findings were confirmed by immunostaining.

**Results:**

In murine models, we identified elevated levels of prostaglandin D_2_ (PGD_2_), prostacyclin, and thromboxanes in neoplasia while prostaglandin E_2_ (PGE_2_), 12-HHTre, HETEs, and HDoHEs are elevated specifically in tumors. Analysis of single-cell RNA sequencing data sets suggests that PGE_2_ and prostacyclins are derived from fibroblasts, PGD_2_, and thromboxanes from myeloid cells, and PGD_2_ and 5-HETE from tuft cells. In patient samples, we identified a transition from PGD_2_- to PGE_2_-producing enzymes in the epithelium during the transition to pancreatic ductal adenocarcinoma, fibroblast/tumor expression of PTGIS, and myeloid/tumor cell expression of TBXAS1.

**Conclusion:**

Our analyses identify key changes in eicosanoid species during pancreatic tumorigenesis and the cell types that contribute to their synthesis. Thromboxane and prostacyclin expression is conserved between animal models and human disease and may represent new druggable targets.

## Introduction

Pancreatic ductal adenocarcinoma (PDAC) can arise from the progression of acinar-to-ductal metaplasia through several grades of pancreatic intraepithelial neoplasia (PanINs), which are characterized by increasing nuclear atypia and loss of cellular polarity. Metaplasia is a process in which acinar cells transdifferentiate into ductal-like cells in response to pancreatic damage to re-establish homeostasis. However, in the context of oncogenic mutations, such as *Kras*^*G12D*^, acinar to ductal metaplasia (ADM) becomes irreversible and leads to the formation of hyperplastic ductal structures known as PanINs. Accumulation of additional genetic mutations leads to tumor formation.^1^ Interestingly, ADM does not result in the formation of a homogeneous population of cells. Instead, ADM generates diverse, differentiated, secretory cells, including tuft cells. Tuft cells, or solitary chemosensory cells, have been detected in chronic pancreatitis and *Kras*^*G12D*^-induced tumorigenesis.^2^^,^^3^ Recently, we showed that tuft cells attenuate pancreatic tumorigenesis through the synthesis and secretion of the eicosanoid prostaglandin D_2_ (PGD_2_).^4^ These cells, which are proposed to play diverse roles in early tumorigenesis and immune cell recruitment, are abundant in preinvasive lesions but are lost in the adenocarcinoma stage of pancreatic tumorigenesis.^2^

Cellular crosstalk within the tumor microenvironment can significantly affect tumor progression. One important class of signaling molecules involved in this crosstalk are eicosanoids, or lipid products derived from the enzymatic or nonenzymatic oxidation of polyunsaturated fatty acids (PUFAs).^5^ Hundreds of eicosanoids have been characterized, each with distinct, context-dependent roles based on the presence and characteristics of receptors expressed in the tumor microenvironment. Eicosanoids can be further subdivided into broad classes—including prostanoids, HETEs, and HdoHEs—based on the precursor molecule PUFA and the synthases responsible for their production.

**Figure 1:**
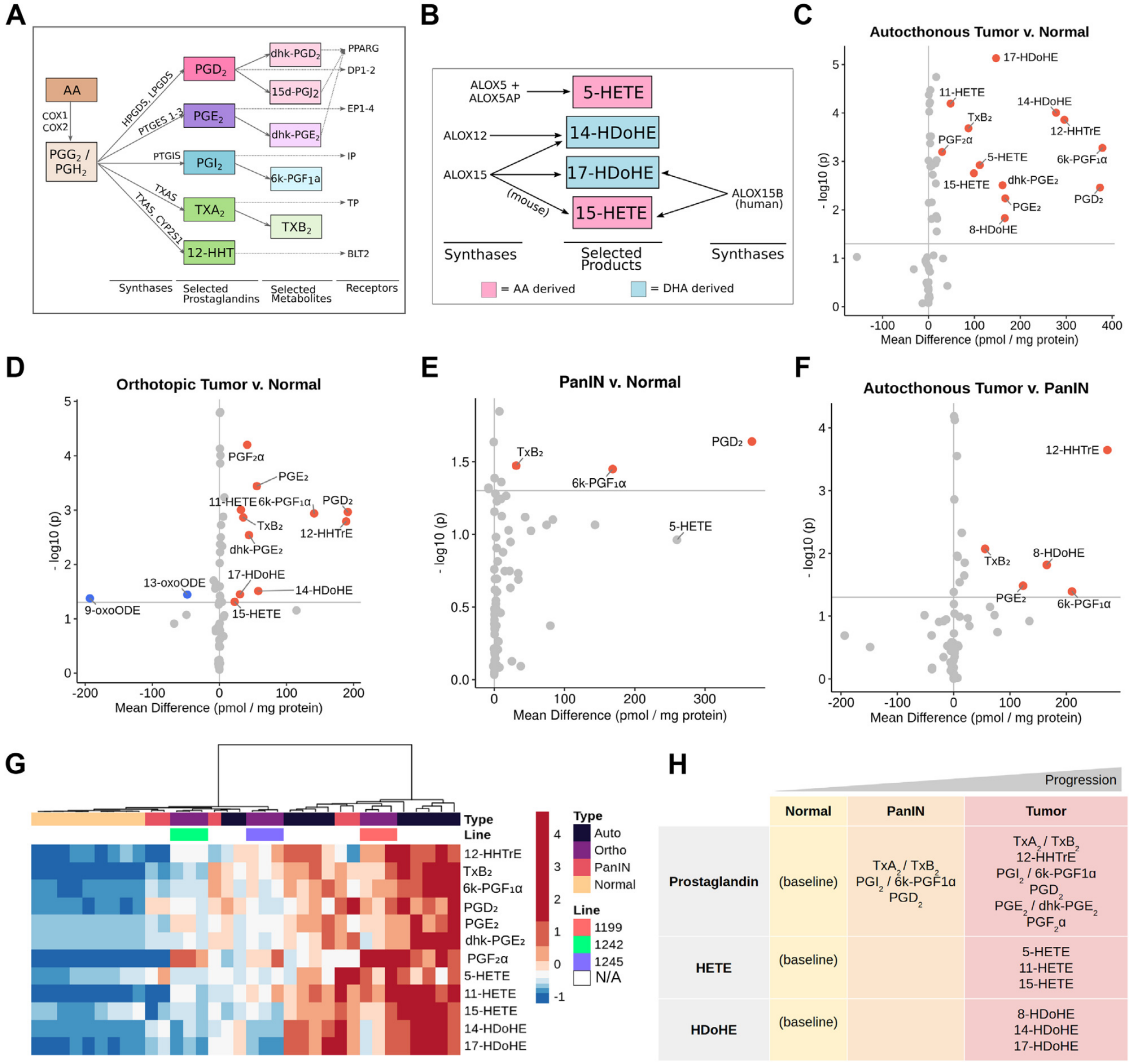
Eicosanoid levels throughout disease progression in mouse models of pancreatic tumorigenesis. Schematics depicting biosynthesis of select (A) prostaglandins and (B) HETEs/HDHEs, along with relevant synthases and receptors. Eicosanoid profiles of (C) autochthonous tumors (*KPC* mice), (D) orthotopic tumors (FC-1199, FC-1242, FC-1245 cell lines), and (E) PanIN-bearing pancreata (*KC* mice) with respect to normal pancreas values. (F) Eicosanoid levels in autochthonous tumors with respect to PanIN. All values, pmol/mg protein. Samples with a mean difference ±20 pmol/mg protein and *P* < .05 are indicated in either red, up, or blue, down. (G) Hierarchical clustering and heatmap of select eicosanoid levels in the samples shown in C–F. Heatmap values are z-score normalized by row, and colors are assigned by quintile scaling. (H) Schematic of major eicosanoids associated with pancreatic tumorigenesis in mouse models.

Arachidonic acid is a common precursor for many different eicosanoids, and certain phospholipases (PLA2G2A, PLA2G4A) can generate arachidonic acid from membrane phospholipids. Synthesis of prostanoid-type eicosanoids begins with metabolism of arachidonic acid by cyclooxygenases (COX1, COX2) to prostaglandin G_2_, which is nonenzymatically metabolized to prostaglandin H_2_. Terminal synthases are required to convert this substrate to specific prostanoid molecules (Figure 1A).^5^^,^^6^

Prostanoids have been studied in the context of tumorigenesis in a variety of organ systems. Prostaglandin E_2_ (PGE_2_) has been linked to tumor growth, metastasis, and fibroblast function in several cancers including PDAC.^5^^,^^7^^,^^8^ Its metabolite, dhk-PGE_2_, is generated by the enzyme HPGD (also known as 15-PGDH); HPGD ablation has been shown to accelerate tumorigenesis in the intestines and pancreas.^9^^,^^10^ PGD_2_ can suppress tumor cell proliferation and metastasis in intestinal adenomas and gastric cancer.^11^^,^^12^ Thromboxane A_2_ (TxA_2_) typically functions as a platelet activator and vasoconstrictor but has also been implicated in promoting tumor cell growth, metastasis, and regulation of neovascularization in breast cancer, lung cancer, and more.^13^, ^14^, ^15^ TxA_2_ has a very short half-life and quickly metabolizes to thromboxane B_2_ (TxB_2_), a product predicted to have minimal biological activity.^16^ Produced in equimolar ratios in thromboxane biosynthesis is prostaglandin 12-HHTre, a relatively understudied eicosanoid with only one known receptor, BLT2 (*LTB4R2*).^17^ 12-HHTre can also be produced by cytochrome proteins, such as CYP2S1.^18^ Although prostacyclin (PGI_2_) antagonizes the actions of TxA_2_ and acts as a vasodilator and inhibitor of platelet activation, it too has been implicated in protumorigenic and proangiogenic roles in the tumor microenvironment and is associated with poor prognosis in lung, ovarian, and gastric cancers.^19^^,^^20^ It also has a short half-life and readily breaks down into the inert, stable product 6k-PGF1α.^21^

Lipoxygenases oxidize arachidonic acid to generate HETEs (Figure 1B).^22^, ^23^, ^24^ 5-HETE is generated from 5-lipoxygenase (ALOX5); ALOX5 inhibition has been shown to slow the growth of cancer cells in vitro.^25^, ^26^, ^27^ The role of eicosanoid 15-HETE, on the other hand, is less clear, as it has been implicated in both protumorigenic and antitumorigenic roles.^28^^,^^29^ Lipoxygenases can metabolize the PUFA docosahexaenoic acid into HDoHEs (14-HDoHE, 17-HDoHE) and biosynthetic precursors to maresins and D-resolvins, respectively.^30^^,^^31^ While the action(s) of HDoHEs in cancer are unknown, their metabolic byproducts can attenuate inflammation and wound healing, and maresins can inhibit tumor growth.^32^

Broad inhibition of prostanoid synthesis by targeting the upstream synthase COX2 has been shown to reduce tumor growth and metastasis, reverse collagen deposition, sensitize tumors to immunotherapy, normalize the vasculature, and enhance the response to chemotherapy in mouse models.^7^^,^^33^^,^^34^ However, COX2 inhibition blocks the synthesis of several downstream eicosanoids, causing significant off-target effects. To develop better, more specific therapeutics, it is important to characterize eicosanoid diversity in PDAC. To accomplish this, we conducted eicosanoid profiling on normal pancreata and PDAC in mouse models of pancreatic tumorigenesis and human patient samples. We then interrogated published single-cell RNA sequencing (scRNA-seq) data sets to identify the cellular source(s) of eicosanoid synthases and receptors and validated key findings at the protein level.

## Methods

### Mice

Mice were housed in accordance with National Institutes of Health guidelines in American Association for Accreditation of Laboratory Animal Care-accredited facilities at the Salk Institute for Biological Studies. The Salk Institute Institutional Animal Care and Use Committee approved all animal studies. *LSL-Kras*^*G12D/+*^, *Ptf1a*^*Cre/+*^, *Pdx1Cre*, *Trp53*^*R17H*^, and *Trp53*^*fl/fl*^ mice have previously been described.^35^^,^^36^ C57B6/J mice were either purchased from the Jackson Laboratory (Bar Harbor, ME) or bred in-house. CD1 mice were either purchased from Charles River Laboratories (Wilmington, MA) or bred in-house.

### Human Samples

Distribution and use of all human samples was approved by the Institutional Review Boards of the Salk Institute for Biological Studies and Vanderbilt University. Flash frozen human pancreas samples were either purchased from Indivumed (Frederick, MD) or were acquired from the Cooperative Human Tissue Network. All normal pancreas samples were acquired from PDAC patients and were pathologically determined to be normal.

### Comprehensive Eicosanoid Panel

Eicosanoid profiling was conducted on flash frozen pancreas tissue from wild-type mice (C57B6/J or CD1), *KPC* mice, or orthotopic tumors (cell lines FC-1199, FC-1242, and FC-1245) grown in C57B6/J mice. In separate experiments, eicosanoid profiling was performed on flash frozen human PDAC or normal pancreas. Age-matched normal and PanIN-bearing pancreata were included from a previous study.^4^ Tissues were homogenized in 1 mL of phosphate buffered saline containing 10% ethanol, and 300 μL was extracted using strata-x polymeric reverse-phase columns (88-S100-UBJ Phenomenex). Samples were taken up in 50 μL of 63% H_2_0%, 37% acetonitrile, and 0.02% acetic acid, and 10 μL was injected into UPLC (ACQUITY ultra performance liquid chromatography System, Waters) and analyzed on a Sciex 6500 Qtrap mass spectrometer at the UCSD Lipidomics Core as previously described.^37^ Tissue eicosanoid concentrations were quantified using deuterated internal standards in conjunction with standard curves obtained in parallel using identical conditions as previously described^38^ and were normalized to total protein mass of the sample.

### Statistical Analysis

Statistical analyses, data processing, heatmap plotting, hierarchical clustering, and principal component analysis were performed in R (https://www.r-project.org/) and/or Prism (GraphPad). Statistical significance was calculated by either 2-tailed unpaired t-tests assuming equal variance or one-way ANOVA. qRT-PCR and immunohistochemistry quantification data are expressed as mean ± standard deviation. Eicosanoid levels are expressed as mean ± standard error of the mean.

## Results

### Eicosanoid Composition Evolves Throughout Pancreatic Tumorigenesis

To broadly profile eicosanoid diversity, we used mass spectrometry and assessed a panel of over 157 eicosanoid species in normal pancreata; autochthonous tumors from either *Kras*^*G12D*^, *Trp53*^*R172H*^, *Ptf1a*^*Cre/+*^ or *Kras*^*G12D*^, *Trp53*^*fl/fl*^, *Pdx1-Cre* mice (collectively referred to as *KPC*); and syngeneic orthotopic tumors generated from 3 separate PDAC cell lines (derived from *KPC* mice) (Figure A1A, File A1). We identified significant increases in mean concentration for several eicosanoids in autochthonous *KPC* tumors (n = 11) compared with normal pancreata (n = 9) (Figure 1C, mean difference > 20 pmol/mg protein, *P* < .05). Most of the elevated species belong to the prostaglandin class of eicosanoids, including PGE_2_ and its metabolite dhk-PGE_2_, PGD_2_, thromboxane synthesis byproducts 12-HHTrE and TxB_2_, and prostacyclin (PGI_2_) metabolite 6k-PGF1α (Figure 1A and C). Other eicosanoids identified in *KPC* tumors are lipoxygenase-derived HETEs (5-HETE, 11-HETE, 15-HETE) and docosahexaenoic acid-derived HDoHEs (14-HDoHE, 17-HDoHE, 8-HDoHE) (Figure 1B and C). To determine if eicosanoid levels in orthotopically generated PDAC tumors reflect those identified in autochthonous models, we analyzed tumors generated from 3 different murine PDAC cell lines (FC-1199, FC-1242, and FC-1245, n = 3/line). Interestingly, most eicosanoids identified as elevated in autochthonous models, relative to normal pancreas, were also elevated in orthotopic tumors except for 5-HETE (Figure 1D). Additionally, analysis of orthotopic tumor profiles identified a significant decrease in linoleic acid oxidation products 9- and 13-oxoODE, as compared to normal pancreas, which did not reach significance in autochthonous PDAC tumor profiles (Figure 1D).

Previously, we profiled eicosanoid species in normal and PanIN-bearing pancreata from 8- to 10-month-old *Kras*^*G12D*^*; Ptf1a*^*Cre/+*^ (*KC*) mice (n = 5) and discovered significant upregulation of several eicosanoids, including TxB_2_, 6k-PGF1α, and PGD_2_; 5-HETE was found to be elevated but did not reach significance (Figure 1E).^4^ To determine how eicosanoid species and levels change between preinvasive PanIN and PDAC, we compared these data to the profiles we generated for *KPC* tumors. We observed a statistically significant gain in prostaglandins TxB_2_, 12-HHTrE, 6k-PGF1α, PGE_2_, and 8-HDoHE in tumors compared with PanIN (Figure 1F). Interestingly, 12-HHTre appears to be tumor-specific. We next examined eicosanoid patterns across all the profiled samples using hierarchical clustering of select eicosanoids (Figure 1G). While normal samples clustered tightly and displayed low levels of selected eicosanoids, murine tumors displayed a large degree of heterogeneity, consistent with heterogeneity observed in tumorigenesis and stromal deposition in these models.^35^^,^^36^ Notably, orthotopic tumors clustered by cell line, with those derived from FC-1199 displaying a trend toward increased eicosanoid production as compared to FC-1242 and FC-1245. While autochthonous tumors displayed an overall higher level of the selected eicosanoids, several samples clustered closer to orthotopic tumor samples. The 5 profiled PanIN samples displayed sample-to-sample heterogeneity, consistent with disease progression in this model, but were characterized by intermediate levels of select eicosanoids (Figure 1G).^39^

Altogether, these analyses identify classes of eicosanoids that are altered during pancreatic tumorigenesis. Specifically, several prostaglandins (PGE_2_, PGD_2_, TxB_2_, 12-HHTrE, 6k-PGF1α), HETEs, and HDoHEs are upregulated in murine tumor models in a relatively consistent manner, although there is heterogeneity within and between commonly used PDAC models (Figure 1H).

### Cell Type-Specific Expression of Eicosanoid Synthases and Receptors in Murine PanIN

In our eicosanoid profiling studies, we identified significant changes in several eicosanoid species during pancreatic tumorigenesis (Figure 1G). As pancreas cellular composition changes with disease progression, the relative contribution of each of these various cell types (ie, epithelium, inflammatory cells, fibroblasts) to eicosanoid production likely changes as well. To identify these changes, we examined available scRNA-seq data sets generated from murine models of PanIN and PDAC. We focused our analyses on eicosanoid synthases and receptors with well-defined biosynthetic pathways, including prostaglandins and lipoxygenases.

**Figure 2:**
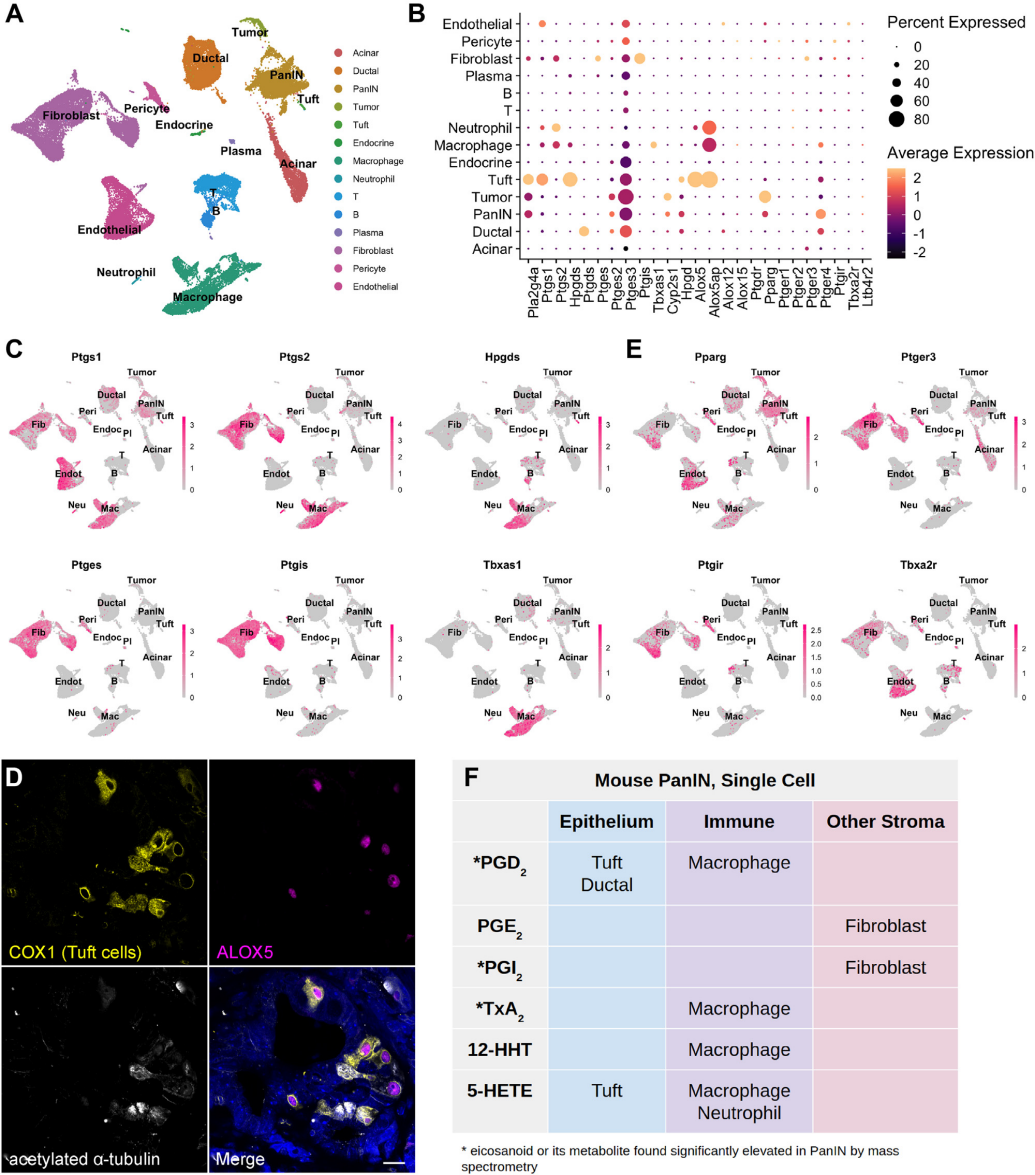
Cell type-specific expression of eicosanoid synthases and receptors in murine PanIN. (A) UMAP of a murine PanIN scRNA-seq data set, generated from the study by Schlesinger et al,^40^ annotated by cell type. (B) Dotplot of average and percent expression of select eicosanoid synthases and receptors in each cell type identified. (C) UMAPs depicting gene expression of select eicosanoid synthases. (D) Co-IF for COX1 (yellow), ALOX5 (magenta), and acetylated α-tubulin (white) in a *KC* pancreas. DAPI, blue. Scale bar, 10 μm. (E) UMAPs depicting gene expression of select eicosanoid receptors. Color intensity in (C) and (E) indicates the normalized gene expression level for a given gene in each cell. (F) Table summarizing inferred cellular sources of eicosanoids based on synthase expression patterns.

Schlesinger et al^40^ recently generated an extensive scRNA-seq data set comprised of 43,897 cells encompassing epithelial and stromal cell types from 9 *Kras*^*G12D*^*;Ptf1aCre*^*ERTM/+*^*;Rosa26*^*LSL-tdTomato/+*^ mice at various stages of disease progression. To identify major cellular sources of eicosanoid synthesis, we reanalyzed the Schlesinger data set. As shown in Figure 2A, sequence libraries were combined, and clusters were annotated by examining both classic cell-type markers and previously published single-cell gene signatures (Figure A1B).^41^ As several time-points were included in this data set, normal pancreas cells, preinvasive cells (including tuft cells), and tumor cells are represented. We first examined expression of upstream synthases in the prostaglandin synthesis pathway. We detected expression of the phospholipase *Pla2g4a* in epithelial cells—including preinvasive cells and tuft cells—as well as in stromal cells such as fibroblasts (Figure 2B). The cyclooxygenases *Ptgs1* (COX1) and *Ptgs2* (COX2) are coexpressed in macrophages, fibroblasts, and tuft cells (as previously described).^2^ *Ptgs1* is additionally expressed in endothelial cells and several immune cell types (Figure 2B and C). We next examined expression of terminal prostaglandin synthases and identified macrophages and tuft cells to be major sources of the PGD_2_ synthase *Hpgds*, while ductal cells strongly express the less efficient PGD_2_ synthase *Ptgds*. All PGE_2_ synthases (*Ptges1-3*) and PGI_2_ synthase *Ptgis* are expressed in fibroblasts. The thromboxane (TxA_2_, TxB_2_) and 12-HHTre synthase *Tbxas1* is detected in macrophages, while 12-HHTre synthase *Cyp2s1* is expressed in PanIN and tumor cells (Figure 2B and C). In terms of lipoxygenase expression, 5-HETE requires coexpression of *Alox5* and its activating protein *Alox5ap*, which are both observed in macrophages, neutrophils, and tuft cells (Figure 2B).^5^ To confirm expression of ALOX5 in tuft cells, we conducted coimmunofluorescence for ALOX5 and COX1 and acetylated-α-tubulin, which label tuft cells in the epithelium. Consistent with studies conducted in the intestines,^42^ we found that 84% (336/400) of tuft cells (n = 4 *KC* mice) express ALOX5. Conversely, 99.5% (398/400) of ALOX5-expressing cells in the epithelium are tuft cells, suggesting a role for pancreatic tuft cells in 5-HETE synthesis (Figure 2D and Table A1).

To determine which cell types might respond to relevant eicosanoids, we evaluated patterns of eicosanoid receptor expression. We identified widespread expression of receptor *Pparg* (PGD_2_ and PGE_2_ metabolites, 15-HETE) in normal and preinvasive ductal cells as well as in several stromal populations. Expression of PGI_2_ receptor *Ptgir* (6k-PGF1α) is concentrated in fibroblasts and pericytes, and the thromboxane receptor *Tbxa2r* (TxB_2_) is highly expressed in fibroblasts, pericytes, and endothelial cells. PGE_2_ receptors were also detected in our analysis, with *Ptger3* expression largely relegated to fibroblasts and *Ptger4* mainly expressed in normal and preinvasive ductal cells, macrophages, T cells, and fibroblasts (Figure 2B and E).

Collectively, our eicosanoid profiling and analysis of the Schlesinger et al scRNA-seq data set suggest potentially critical cellular sources of key eicosanoids in preinvasive PanIN. For example, high levels of PGD_2_, PGI_2_ metabolites (6k-PGF1α), and TxA_2_ metabolites (TxB_2_) by mass spectrometry may be explained by the expression of requisite synthases in tuft cells and macrophages, fibroblasts, and macrophages, respectively (Figure 2B, C and F).

### Expression of Eicosanoid synthases and receptors in murine models of PDAC

**Figure 3:**
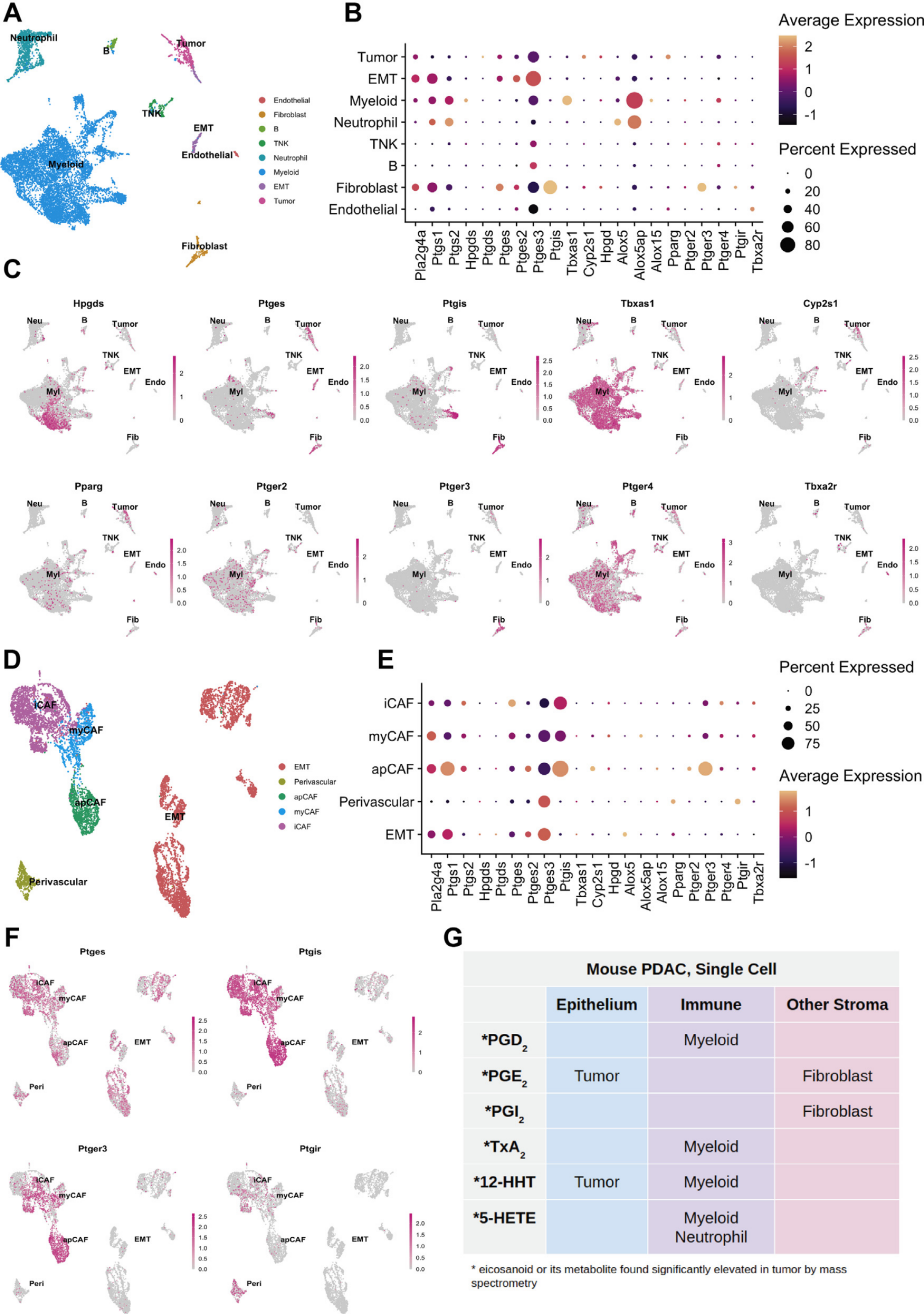
Cell type-specific expression of eicosanoid synthases and receptors in murine models of PDAC. (A) UMAP of a murine PDAC (*KPC*) scRNA-seq data set derived from the study by Elyada et al, annotated by cell type. (B) Dotplot of average and percent expression of select eicosanoid synthases and receptors in each cell type. (C) UMAPs depicting gene expression of select eicosanoid synthases/receptors. (D) UMAP of a FACS-enriched CAF data set derived from the study by Elyada et al, annotated by cell type. (E) Dotplot of average and percent expression of select eicosanoid synthases and receptors in each cell type. (F) UMAPs depicting gene expression of select eicosanoid synthases/receptors. Color intensity in (C) and (F) indicates the normalized gene expression level for a given gene in each cell. (G) Table summarizing inferred cellular sources of eicosanoids based on patterns of synthase expression. apCAF, antigen presenting CAF; EMT, epithelial to mesenchymal transition; iCAF, Inflammatory cancer-associated fibroblast; myCAF, myofibroblastic CAF; TNK, T and natural killer cell.

To examine eicosanoid synthase and receptor expression in murine PDAC, we reanalyzed a scRNA-seq data set generated from *KPC* mice (Figure 3A).^41^ This data set is comprised of 11,222 cells, and libraries were combined and clusters annotated by expression of known cell type markers (Figure A1C). In contrast to the Schlesinger data set, epithelial clusters in this data set are largely comprised of tumor cells (Figure 3A). In terms of eicosanoid synthases, we identified expression of phospholipase *Pla2g4a* in tumor cells, fibroblasts, and myeloid cells, with minor expression in neutrophils (Figure 3B). Cyclooxygenases (*Ptgs1*, *Ptgs2*) are expressed primarily in epithelial to mesenchymal transition-like tumor cells, myeloid cells, neutrophils, and fibroblasts (Figure 3B). We identified the full complement of genes required to produce PGD_2_ (*Hpgds*); thromboxane pathway products TxB_2_ and 12-HHTre (*Tbxas1*); and 5-HETE (*Alox5*, *Alox5ap*), as well as *Alox15* (15-HETE and 14- and 17-HdoHE) in myeloid cells. Tumor cells express genes required to produce PGE_2_ (*Ptges*) and 12-HHTre (*Cyp2s1*). Although few fibroblasts were profiled in this data set, enzymes required to produce PGE_2_ and PGI_2_ (*Ptgis*) were detected. Finally, neutrophils express enzymes required for 5-HETE synthesis (*Alox5*, *Alox5ap*) (Figure 3B and C).

We next examined expression of known eicosanoid receptors. *Pparg*, which mediates the effects of several eicosanoids, is most highly expressed in tumor cells and myeloid cells.^5^ PGE_2_ receptors are heterogeneously expressed in myeloid cells, tumor cells, neutrophils, and fibroblasts. While this data set encompasses only a small number of endothelial cells, we could readily detect expression of both *Pparg* and thromboxane receptor *Tbxa2r*, consistent with the Schlesinger data set (Figure 3B and C). To collectively validate our findings in the PanIN and PDAC data sets, we reanalyzed a third scRNA-seq data set that encompasses normal pancreas, preinvasive disease, and PDAC in multiple murine models (Figure A2).^43^ Several patterns found in the Schlesinger data set were identified here as well, notably the presence of *Ptgds* and absence of *Ptges* in the epithelium in early stages of disease progression. Analyses of PDAC are largely in agreement with observations made from the Elyada et al^41^ data set, including expression of PGE_2_ synthases in tumor cells, *Ptgis* in fibroblasts, and *Tbxas1* in macrophages/myeloid cells (Figure A2).

Cancer-associated fibroblasts (CAFs) have been established as important players in cancer formation and progression, and several subsets have been identified in PDAC.^41^^,^^44^ To determine if eicosanoid synthases and/or receptors are differentially expressed in various CAF subtypes, we re-examined a second scRNA-seq data set from Elyada et al,^41^ consisting of FACS-enriched fibroblasts. This data set is composed of 8438 cells generated from 4 tumor-bearing *KPC* mice and includes 3 CAF subtypes (iCAFs, myCAFs, and apCAFs), perivascular cells, and EpCAM-negative epithelial to mesenchymal transition-like tumor cells, as annotated by the original authors (Figure 3D, Figure A1D). We found eicosanoid synthase and receptor expression in this fibroblast-enriched data set to be largely consistent with our previous analyses, showing that fibroblasts express *Pla2g4a*, *Ptgs1* (COX1), PGE_2_ synthases, and *Ptgis*. Fibroblasts specifically express PGE_2_ receptor *Ptger3*, which is highest in apCAFs. Interestingly, the PGI_2_ (6k-PGF1α) receptor, *Ptgir*, is also enriched in perivascular cells suggesting a possible signaling loop between CAFs and perivascular cells (Figure 3E and F).

To evaluate whether orthotopic tumor models recapitulate the patterns of eicosanoid synthase expression we identified in *KPC* mice, we generated tumors using the FC-1199 and FC-1245 PDAC cell lines. Tumors were collected, and epithelial (EpCAM + CD45−) or inflammatory (EpCAM-CD45+) cells were isolated by FACS. Separately, RNA was collected from chunks of whole tumor or cell lines grown in 2-D. We then compared expression of eicosanoid synthases between these different models and tissue compartments by qRT-PCR and immunofluorescence. In tumors derived from both cell lines, we found eicosanoid synthases (*Ptgs1*, *Ptgs2*, *Hpgds*, *Alox5*) to be more highly expressed in immune cells than in tumor cells although expression is not exclusive (Figure A3A and B; Table A2). PGD_2_ synthase *Ptgds* and PGE_2_ synthases *Ptges1-3* are also expressed in tumor cells (Figure A3A and B). Fibroblasts are less abundant in these tumors than in autochthonous models, but we were able to identify expression of PTGIS in αSMA + fibroblasts by immunofluorescence (Figure A3C). Notably, and in comparison to autochthonous models, eicosanoid synthesis in orthotopic models appears to be dominated by the stromal compartment rather than the tumor cells (Figure A3).

**Figure 4:**
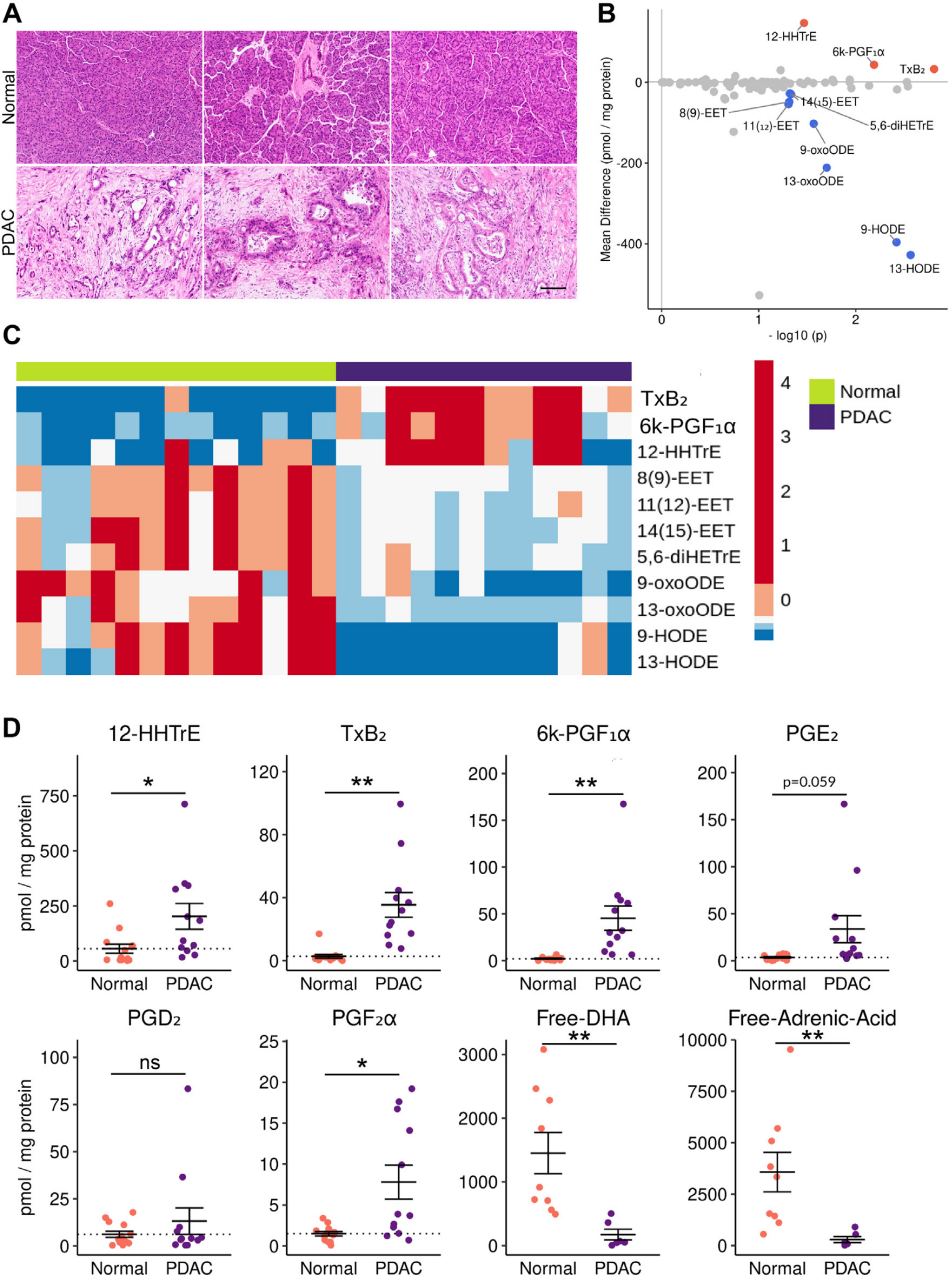
Eicosanoid profiling of human PDAC. (A) Representative H&E of normal human pancreata and PDAC samples. Scale bar, 100 μm. (B) Comparison of normal pancreas and PDAC eicosanoid profiles (pmol/mg protein). Samples with a mean difference > +/−20 pmol/mg protein and *P* < .05 are indicated in either red, up, or blue, down. (C) Heatmap of select eicosanoids from normal pancreata and PDAC. Values are z-score normalized by row, and colors are assigned by quintile scaling. (D) Dotplots of select eicosanoids. Error bars, standard error of the mean. ∗*P* < .05; ∗∗*P* < .01; ∗∗∗*P* < .001; ∗∗∗∗*P* < .0001.

**Figure 5:**
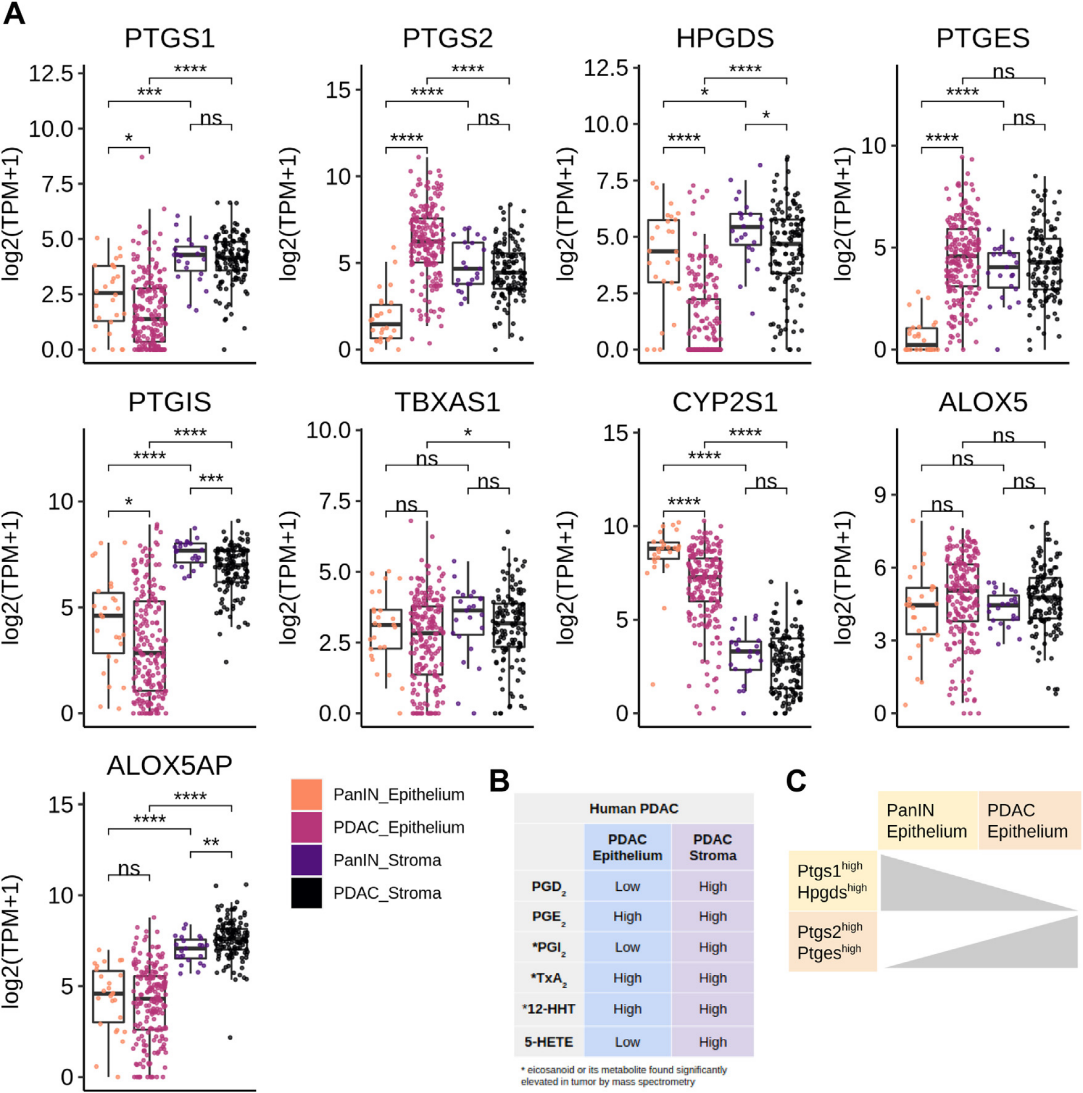
Eicosanoid synthase expression in human PanIN and PDAC. (A) Boxplots comparing expression (log2 (TPM + 1)) of microdissected stroma and epithelium from PanIN (n = 26) and PDAC (n = 197 epithelium, 124 stroma). ∗*P* < .05; ∗∗*P* < .01; ∗∗∗*P* < .001; ∗∗∗∗*P* < .0001. (B) Qualitative summary describing relative localization of terminal eicosanoid synthases in either the tumor epithelium or stroma. (C) Summary schematic describing a switch in prostaglandin synthase expression in the epithelium transitioning from PanIN to PDAC.

**Figure 6:**
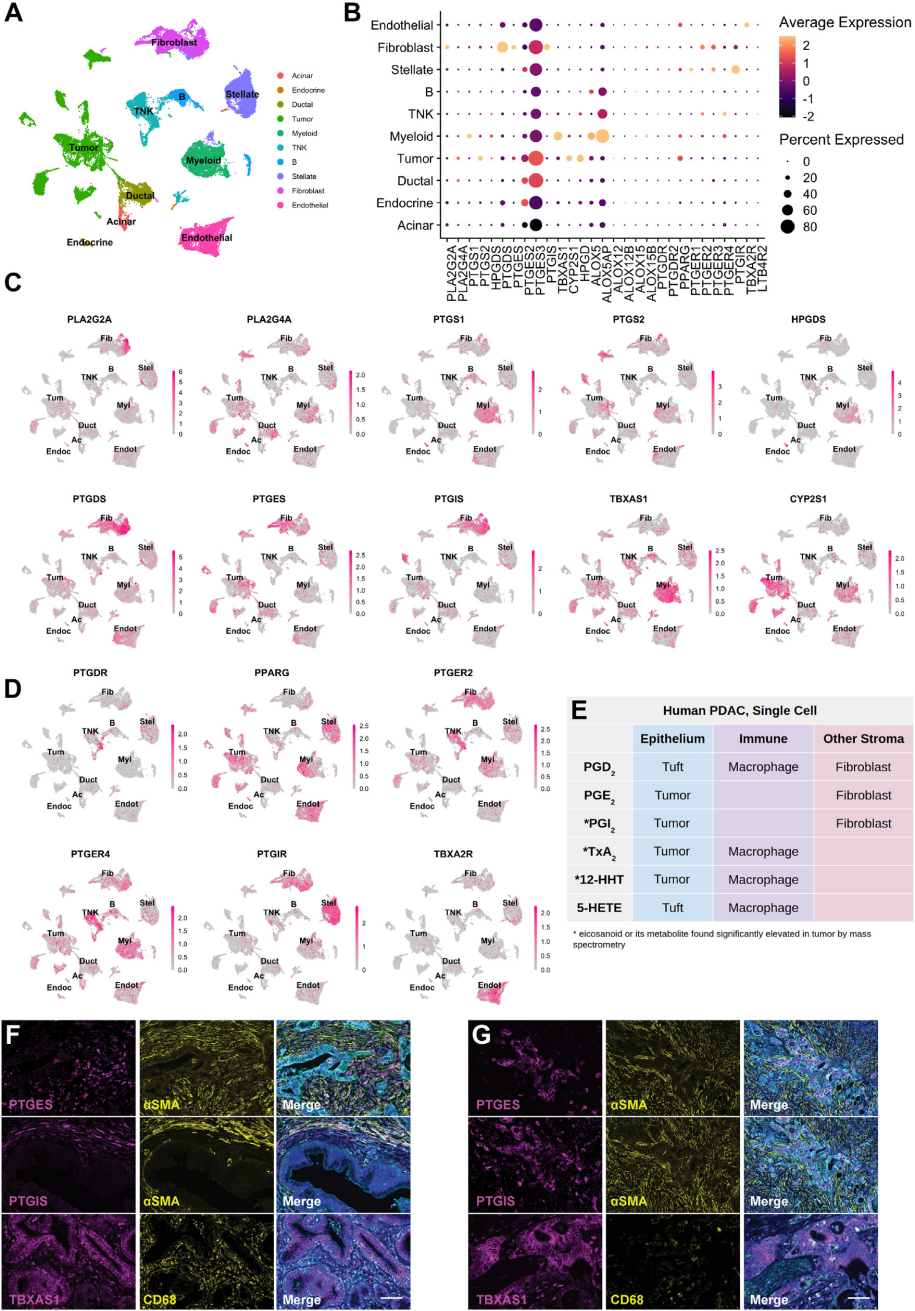
Cell type-specific expression of eicosanoid synthases and receptors in human PDAC. (A) UMAP of a human PDAC scRNA-seq data set generated by Peng et al, subsetted to exclude adjacent normal samples and annotated by cell type. TNK, T and natural killer cells. (B) Dotplot of average and percent expression of select eicosanoid synthases/receptors in each cell type. UMAPs of either eicosanoid (C) synthase or (D) receptor gene expression. Color intensity indicates the normalized gene expression level for a given gene in each cell. (E) Table summarizing inferred cellular sources of eicosanoids based on patterns of synthase expression. (F) Co-immunofluorescence for relevant eicosanoid synthases (magenta), stromal markers (αSMA, fibroblasts; CD68, macrophages, yellow), and γactin (blue) highlighting stromal or (G) tumor expression. Scale bar, 50 μm.

**Figure 7:**
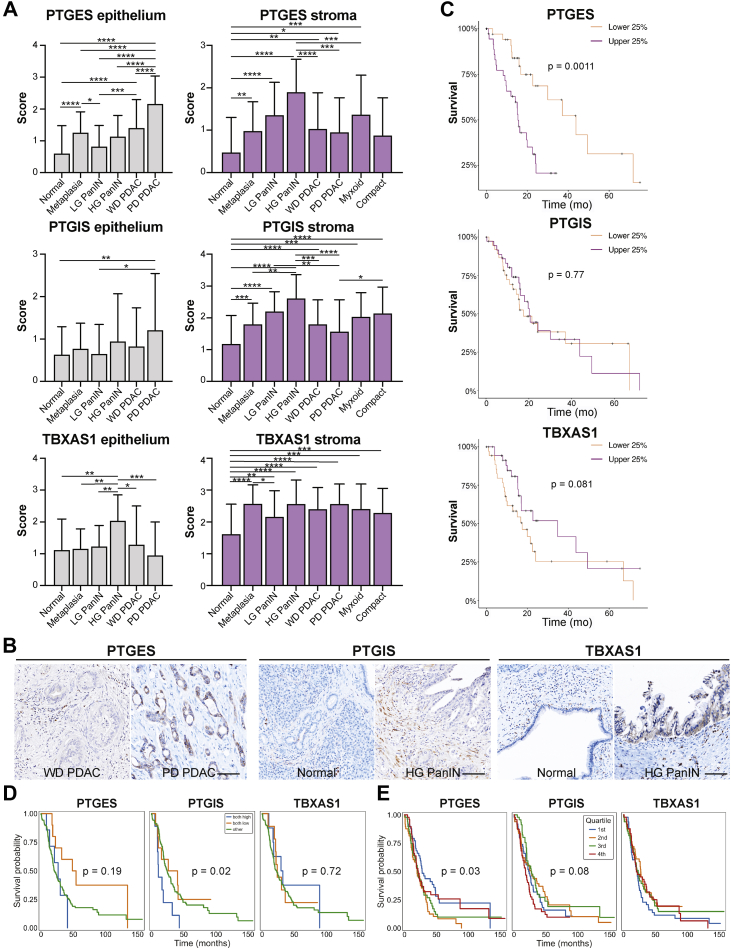
Localization and survival benefit for select eicosanoid synthases in human PDAC. (A) Quantification of scored IHC for PTGES, PTGIS, or TBXAS1 from 22 patients encompassing normal, PanIN-, and PDAC-associated stroma and epithelium as well as both myxoid and compact fibrosis. (B) Representative IHC images. Scale bar, 100 μm. (C) Survival curves for total PTGES, PTGIS, or TBXAS1 expression generated from the TCGA database, representing 150 PDAC patients. The top and bottom 38 patients are shown. Survival curves for (D) total (epithelial and stromal expression combined where both high means high expression in both compartments) or (E) epithelium-specific PTGES, PTGIS, or TBXAS1 expression generated from the Maurer et al data set representing 197 epithelial samples and 124 stromal samples from PDAC patients. Quartiles reflect expression broken out into 25% of patients ranked by expression level of a given synthase.

### Eicosanoid Profiling of Human PDAC

To determine if the eicosanoid profiles we generated from murine PDAC are representative of the human condition, we next conducted mass spectrometry on surgical specimens collected from 12 patients with PDAC, as well as 13 pancreas samples pathologically determined to be normal (Figure 4A, File A1). Eicosanoid profiling of human samples revealed large, significant upregulation of prostaglandins in the thromboxane pathway (TxB_2_, 12-HHTre) and the prostacyclin pathway (6k-PGF1α), consistent with observations made in mouse models (Figure 1). Although it did not reach significance, we did see a trend toward increased PGE_2_ levels in PDAC (*P* = .059), in agreement with previous studies (Figure 4D).^7^ Consistent with our observations in murine orthotopic tumor models, we observed a significant decrease in multiple linoleic acid metabolites in PDAC as compared to normal pancreas (Figure 4B–D). We also detected a significant decrease in free adrenic acid and docosahexaenoic acid (Figure 4D). Interestingly, and in agreement with our previous studies of HPGDS expression, we did not detect high levels of PGD_2_ (Figure 4D).^4^ While this may be representative of human PDAC, we cannot exclude confounding factors, such as sample preparation. The lack of detection of additional eicosanoids found to be elevated in mouse models may be due, in part, to degradation.

### Eicosanoid Gene Expression Changes During the Transition From Preinvasive PanIN to PDAC

As our eicosanoid profiling analysis of human PDAC identified several species that are also upregulated in mouse models, we next asked whether the cellular sources of these relevant eicosanoid synthases are the same and if they change during the PanIN to PDAC transition. To answer these questions, we examined a bulk RNA sequencing data set of laser capture microdissected samples of human epithelium and stroma from 26 patients with PanIN and 197 patients with PDAC (124 matched stromal samples, generated as described in the study by Maurer et al^45^). First, we examined cyclooxygenase gene expression patterns. We found that while *PTGS1* (COX1) expression is higher in the stroma than in the epithelium in both PanIN and PDAC, there is a significant decrease in gene expression in the epithelium from PanIN to PDAC, consistent with tuft cell loss (Figure 5A).^2^ Interestingly, there is a gain in *PTGS2* (COX2) expression in the epithelium in the transition to PDAC, and levels are higher than those in the stoma, consistent with a role for tumor cell-derived COX2 (Figure 5A).

Next, we examined expression patterns of terminal prostaglandin synthases. While both PGD_2_ synthase *HPGDS* and PGE_2_ synthase *PTGES* are expressed in the stroma of PanIN and PDAC, we found a large, significant decrease in *HPGDS* and a gain of *PTGES* expression in the PDAC epithelium (Figure 5A–C). These results suggest a possible switch between tumor suppressive PGD_2_ and protumorigenic PGE_2_ during the transition from PanIN to PDAC.

Other features of human PDAC identified in this analysis are consistent with what we found in mouse models, namely higher expression of *PTGIS* in the stroma and *CYP2S1* in the epithelium (Figure 5A). Thromboxane synthase *TXBAS1* was detected at comparable levels in the epithelium of both PanIN and PDAC but is significantly higher in the PDAC stroma, consistent with the increase in TxB_2_ levels we found between PanIN and PDAC in mouse models (Figure 5A). While *ALOX5* is expressed at comparable levels in all conditions, *ALOX5AP* is elevated only in stromal samples (Figure 5A). Taken together, these data show that the cell type-specific expression patterns of eicosanoid synthases in human PanIN and PDAC largely agree with those in mouse models although tumor cells may play a larger role in human disease (Figure 5B, Figure A4). Over the course of pancreatic tumorigenesis, we observed a shift in the epithelium from *PTGS1*/*HPGDS* to *PTGS2*/*PTGES* expression (Figure 5C). This is consistent with our observation that *PTGS1* + *HPGDS* + tuft cells are lost in the transition from preinvasive disease to PDAC.^2^

### Cell Type-Specific Expression of Eicosanoid Synthases and Receptors in Human PDAC

To identify the specific epithelial and stromal cell types that express relevant eicosanoid synthases and receptors in human PDAC, we next examined a scRNA-seq data set of 11 normal pancreata and 24 PDAC samples including 57,530 cells.^46^ We first subsetted the tumor samples (41,986 cells) and identified cell types by markers described in the study of Peng et al (Figure 6A).^46^ Interestingly, we identified a large degree of heterogeneity in synthase expression among malignant tumor cells, possibly reflecting the intertumor heterogeneity of patients included in this study (Figure 6B and C). Within the tumor population, we identified expression of phospholipase enzymes *PLA2G2A* and *PLA2G4A* and *PTGS2* (COX2), but not *PTGS1* (COX1) (Figure 6B and C). Tumor cells express synthases *TBXAS1* (TXA_2_, TXB_2_, 12-HHTre) and *CYP2S1* (12-HHTre), and some subclusters are enriched for PGE_2_ synthase *PTGES* or prostacyclin synthase *PTGIS* (Figure 6B and C). A small subset of 49 cells that cluster with ductal cells are tuft cells and express both *PTGS1* and *HPGDS*, as previously described (Figure A5A).^2^ In terms of the stroma, we identified expression of *PTGS1*, *TBXAS1*, and *CYP2S1* enriched in macrophages. A subset of these cells also weakly express *HPGDS*. PDAC CAFs express *PTGDS* (PGD_2_), *PTGES* (PGE_2_), and *PTGIS* (PGI_2_) (Figure 6B and C). *ALOX5/ALOX5AP* (5-HETE) coexpression is largely detected in myeloid cells, but also in B cells. Consistent with the Maurer data set, *ALOX15B* (15-HETE, 17-HdoHE) is expressed in myeloid cells and fibroblasts (Figure 6B, Figure A4).^45^ To confirm these data, we interrogated a second scRNA-seq data set of human PDAC containing 8000 cells and 10 patients.^47^ As shown in Figure A5B, we found that eicosanoid synthase expression patterns in these 2 data sets are largely in agreement.^46^

To infer what cell types might respond to eicosanoids in the PDAC microenvironment, we next examined expression of relevant receptors. *PPARG* (PGD_2_ and PGE_2_ metabolites, 15-HETE) expression is widespread and can be found in tumor cells, myeloid cells, stellate cells, and endothelial cells, with minor expression in several other cell types. *PTGER2* and *PTGER4* share similar patterns of expression, highest in myeloid cells, T cells, and fibroblasts, while *PTGER3* is specifically expressed in stellate cells and fibroblasts. PGD_2_ receptor *PTGDR* is expressed in T cells, fibroblasts, and stellate cells. As seen in murine models, *PTGIR* (PGI_2_, 6k-PGF1α) is highly expressed in stellate cells and fibroblasts, and *TBXA2R* (TxB_2_, TxA_2_) is most highly expressed in endothelial cells (Figure 6D).

To reconcile inferences made from the RNA-seq studies with eicosanoids detected by mass spectrometry, we conducted immunostaining for PTGIS, PTGES, and TBXAS1 in several tissue samples spanning normal pancreas, PanIN, and PDAC. Consistent with sequencing studies, we identified expression of PTGIS and PTGES in both αSMA + fibroblasts and in tumor cells. TBXAS1 is expressed in both CD68 + macrophages and the tumor epithelium (Figure 6F and G).

To take a more quantitative approach, we next conducted immunohistochemistry and scored expression of these synthases in multiple tissue compartments of samples from 22 treatment-naive PDAC patients. By sampling multiple surgical samples from the same patients, our analysis spanned normal tissue, metaplasia, high- and low-grade PanIN, well and poorly differentiated PDAC, and myxoid or compact stroma totaling 1176 regions of interest (Figure 7A, File A2). We note several observations from these analyses, including a significant increase in PTGES expression in the epithelium with disease progression, as predicted in Figure 5, highest in poorly differentiated PDAC. We also identified a significant increase in PTGIS in the stroma and in TBXAS1 in the epithelium of high-grade PanIN, as compared to normal tissue (Figure 7A and B). Using the same data, Spearman Rho correlation analyses between all the datapoint classes identify significant positive and inverse correlations mostly in interrogated normal duct areas (Figure A6A). Notably, PTGIS, PTGES, and TBXAS1 show positively correlated expression in both epithelial and stromal components (*P* = .005–.003 × 10^−5^), while PTGIS and TBXAS1 have an inverse correlation in normal duct epithelium (*P* = .004). Furthermore, the stroma underlying poorly differentiated PDAC showed an inverse relationship between PTGIS and PTGES (*P* = .032).

Finally, to determine if synthase expression correlates to patient survival, we interrogated the TCGA database (150 PDAC patients) as well as the laser-captured RNA-seq data set (Maurer et al, manuscript in preparation, 197 PDAC epithelium, 124 PDAC stroma samples) described in Figure 5. As shown in Figure 7, we identified a survival advantage for patients with low PTGES expression in both data sets and for patients with low PTGIS expression in the Maurer data set (Figure 7C and D). Interestingly, it is tumor cell, and not stromal, expression of PTGES that is associated with a survival advantage in the Maurer data set (Figure 7D and E, Figure A5B). This may reflect different functions for fibroblast vs tumor cell-derived PGE_2_ in PDAC. Collectively, these data suggest that tumor cell expression of eicosanoid synthases is associated with pathogenesis and reduced patient survival.

## Discussion

In this study, we conducted eicosanoid profiling on normal pancreata and PDAC in mouse models of pancreatic tumorigenesis and patient samples, interrogated published RNA-seq data sets to generate predictions as to the cellular origin of eicosanoid species, and validated key findings at the protein level. The data collectively suggest a previously undescribed role for prostacyclin and thromboxane signaling in pancreatic tumorigenesis. Both prostacyclins and thromboxanes have known roles in blood clotting. The incidence of thrombosis is particularly high in patients with PDAC (up to 57%), and thromboembolic diseases are the second most common cause for mortality, accounting for 44% of total deaths after cancer progression.^48^^,^^49^ These data indicate that targeting prostacyclin or thromboxane signaling pathways in PDAC may have the added benefit of inhibiting thrombotic events.

Transcriptomic data from both human and mouse samples suggest an “eicosanoid switch” in the epithelium, from PGD_2_-producing enzymes in PanIN to PGE_2_-producing enzymes in PDAC. Our analysis of the human condition suggests that this gain in synthase expression in tumor cells is associated with reduced survival. In mouse models, the stroma appears to be the dominant source of eicosanoid synthesis, a potentially critical difference between mouse models and human disease. These analyses provide a valuable resource for further functional investigation.

We note certain limitations in the conclusions drawn from the analytical techniques we have used. Although eicosanoid profiling by mass spectrometry informed us of targets highly upregulated in PDAC, the detection of these species may have been impacted by their half-lives. For example, while it can be hypothesized that the relative lack of diversity in eicosanoid species in human PDAC may be a result of years-long immune reprogramming to an immunosuppressed state, in contrast to the months-long time course of tumor progression in mouse models, an equally viable hypothesis is that this lack of eicosanoid diversity is merely a technical artifact arising from sample processing after surgery. This differs from the relatively quick preservation of mouse tumor samples. In support of the latter hypothesis is the fact that the 3 significantly upregulated eicosanoids—or their metabolites—in human tumors have relatively long half-lives. However, the trend toward higher PGE_2_ levels, which has a half-life of only 2.5–5 minutes may serve as a positive control.^50^ These hypotheses may be resolved by accurate *in situ* studies aimed at identifying synthase functionality.

RNA-sequencing-based analyses also present key limitations. RNA expression does not always correlate with protein expression or activity, the latter of which is largely dependent on post-transcriptional factors, post-translational factors, enzyme localization and substrate availability, and signaling cues. Second, the scRNA-seq data sets we used to localize eicosanoid synthase/receptor expression are unsuitable to study platelets, whose small size and lack of a nucleus excluded them from collection. Platelets synthesize eicosanoids (PGE_2_, PGD_2_, 11-HETE, 15-HETE, and others) in various contexts, such as wound healing, and tumor-infiltrating platelets have been described in pancreatic neuroendocrine tumors.^51^^,^^52^ Activated platelets could represent a major source of eicosanoid diversity uncharacterized by our analyses.

Despite these limitations, our analyses identify eicosanoids that warrant further investigation in the context of pancreatic tumorigenesis. Our multifaceted approach enabled us to make novel predictions regarding patterns of eicosanoid synthesis in PanIN and PDAC. We have confirmed these predictions at the synthase RNA, synthase protein, and eicosanoid levels. Notably, many patterns of eicosanoid synthase expression identified in this study are consistent with previous studies in PDAC or in other organ systems. Collectively, our work localizes eicosanoid synthases to specific cell types in PDAC and underscores the need to determine the function of these species in pancreatic cancer development and progression. Understanding the role of various eicosanoids in PDAC may identify pathways to co-opt or novel targets for treatment.

## Supplementary Materials

File A1

File A2

Supplemental Methods

Table A1

Table A2

Figure A1

Figure A2

Figure A3

Figure A4

Figure A5

Figure A6
