# Supplementary material for: Eicosanoids in the Pancreatic Tumor Microenvironment—A Multicellular, Multifaceted Progression
Source: Gastro Hep Adv. 2022 Jun 11;1(4):682–97. doi: 10.1016/j.gastha.2022.02.007 (PMC9583893; doi:10.1016/j.gastha.2022.02.007)
Supplement: Supplemental Methods [file mmc3.docx]

**Supplemental Methods**

**Orthotopic Transplantation.** Cells lines (FC-1199, FC-1242, and FC-1245)^1^ were cultured in 10% FBS (Biowest) and 1% Anti-Anti (Gibco) in DMEM (Gibco). Medium was replaced every three days and plates were split at a 1:4 ratio when reaching 80% confluency. To prepare for transplantation, cells were dissociated with 0.05% Trypsin (Gibco) for 2-4 minutes, and the enzyme was quenched with culture media. Cells were diluted to 100 cells per 20 µl of 1:1 Matrigel (Corning):medium and placed on ice until transplantation. Mice were administered anaesthesia and analgesics in accordance with IACUC protocol. A patch of skin over the spleen was shaved, disinfected with Nolvasan (Patterson Veterinary Supply), and cut to expose the spleen. The pancreas was exposed via gripping the spleen with circular forceps. One hundred cells resuspended in Matrigel:medium were injected into the pancreas using an insulin syringe. Organs were replaced, the peritoneum was sutured, and the skin was stapled. The mice were allowed to recover and were monitored in accordance with IACUC protocol.

**Histological staining.** Tissues were fixed overnight in zinc-containing, neutral-buffered formalin (Fisher Scientific), embedded in paraffin, cut in 5 µm sections, mounted, and stained. Sections were deparaffinized in xylene, rehydrated in a series of graded ethanols, and then washed in PBST and PBS. Endogenous peroxidase activity was blocked with a 1:50 solution of 30% H_2_O_2_:PBS followed by microwave antigen retrieval in 100 mM sodium citrate, pH 6.0. Sections were blocked with 1% bovine serum albumin and 5% normal goat serum in 10 mM Tris (pH 7.4), 100 mM MgCl_2_, and 0.5% Tween-20 for 1hr at room temperature. Primary antibodies (Table S1) were diluted in blocking solution and were incubated on tissue sections overnight. Slides were then washed, incubated in streptavidin-conjugated secondaries (Abcam) and developed with DAB substrate (Vector). Hematoxylin and eosin (H&E) staining was performed to assess tissue morphology. Immunofluorescence on paraffin-embedded tissues followed the immunohistochemistry protocol until the blocking step. Instead, tissues were blocked with 5% normal donkey serum and 1% BSA in 10 mM PBS for 1 hour at room temperature. Tissue sections were stained with primary antibodies in 10 mM PBS supplemented with 1% BSA and 0.1% Triton X-100 overnight. Sections were then washed 3 x 15 min in PBS with 1% Triton X-100, incubated in Alexa Fluor secondary antibodies, washed again for 3 x 5 min, rinsed with distilled water, and mounted with Prolong Gold containing DAPI (Invitrogen). All slides were scanned and imaged on an Olympus VS-200 Virtual Slide Scanning microscope.

**Immunohistological analysis and scoring of patient samples.** H&E and immunohistochemistry of PTGES, PTGIS, and TBXAS was conducted on serial tissue sections obtained from 22 treatment naïve PDAC patients (34 slides total) and was then analyzed with QuPath version 0.3.0, an open-source software for digital pathology and whole-slide image (WSI) analysis, as previously described ^2, 3^. Representative H&E WSI were registered with serial sections of PTGIS, PTGES, and TBXAS1 immunostaining using the “Interactive Image Alignment” extension. Up to three 500 µm x 500 µm regions of interest (normal ducts, acinar-to-ductal metaplasia, low-grade PanIN, high-grade PanIN, well-differentiated PDAC, poorly-differentiated PDAC, myxoid fibrosis and compact fibrosis) were selected on the H&E slide, and the same registered area of the immunostaining was scored by two observers, including one board-certified pathologist subspecialized in gastrointestinal diseases including pancreas (V.Q.T). The epithelial and stromal compartments were scored separately for each component. The scoring scheme for the epithelium was: 0=negative or very rare cells, 1=mild staining in less than 50% of cells, 2=intermediate, 3=more than 50% of cells staining strongly. The scoring scheme for the stromal compartment was: 0=negative or very rare cells, 1=few interspaced cells with mild staining, 2=intermediate, 3=frequent approximated cells with strong staining together.

**Tumor dissociation and fluorescence activated cell sorting (FACS).**Orthotopic tumors were harvested near endpoint (4 weeks for FC-1245, 6 weeks for FC-1199), washed twice with DMEM (Gibco), chopped, and dissociated with 200 mg DNASE I (Sigma-Aldrich), 0.2 mg Pronase (Sigma Aldrich), and 1 mg Collagenase P (Sigma Aldrich) in 10 ml Gey’s solution (Sigma Aldrich) for 50 minutes at 37°C. Cells were passed through a 100 μm filter and washed with 2% FBS (Biowest) in HBSS (Gibco). Cells were incubated with 1 ml ACK lysis buffer (Gibco) for 3 minutes on ice and were then washed twice. Cells were resuspended in 500 μm 2% FBS in HBSS and incubated on ice in the dark with 1 μl Fc block (BD Biosciences) for 3 minutes. Cells were then stained with CD45 (APC, Biolegend) and EpCAM (PE/Cy7, Biolegend), resuspended in 2% FBS in HBSS containing 5μg/ml DAPI (Invitrogen), and sorted into DMEM containing 10% FBS. FACS was conducted at the Salk Institute’s Flow Cytometry Core facility on an Aria Fusion cell sorter (100-µm size nozzle, 1 x PBS sheath buffer with sheath pressure set to 20 PSI). Cells were sorted in 1-drop Single Cell sort mode for counting accuracy. EpCAM+;CD45- cells were sorted as the epithelial fraction and EpCAM- CD45+ cells were sorted as the immune cell fraction

**RNA isolation and qRT-PCR.** Cells were lysed with 1% beta-mercaptoethanol (Sigma) in RLT plus buffer (Qiagen), and frozen at -80°C. Samples were thawed, and RNA was isolated with the Qiagen RNeasy Micro Kit (Qiagen, 74004) according to the manufacturer’s instructions. cDNA synthesis was carried out using iScript reagent (Bio-Rad), and RT-qPCR was performed using Power SYBR Green PCR Master Mix (Applied biosystems) on the ABI 7900 detection system (Applied Biosystems). Relative expression values were determined using the standard curve method and were normalized to housekeeping gene Rplp0. Primer sequences can be found in Table S2.

**Bulk Human RNA Sequencing Analysis.** Compartment-specific gene expression profiles of human PanIN (n = 26) and PDAC (n = 197) were generated using laser capture microdissection with subsequent RNA sequencing as previously described ^4, 5^. Statistics were computed with DESeq2. To study the association of epithelial, stromal or joint eicosanoid enzyme expression, respectively, and patient outcome, we binned our patient cohort (n=197 for epithelial and n=124 for stromal and joint evaluation) into quartiles according to the expression of the respective enzyme and compared differences in outcome between the upper and lower quartile using a log-rank test as implemented in logrank_test function from the lifelines Python package (Davidson-Pilon et al., 2021).

**Single Cell RNA Sequencing Analysis.** Processed count matrices for scRNA-seq datasets were downloaded from the Gene Expression Omnibus (GEO) database ^6-8^. Quality controls for read counts, genes expressed, and mitochondrial gene expression are described in the respective publications. Normalization, variable feature selection, and scaling were performed with Seurat ^9^. Data preprocessing and dimensionality reduction was performed with Seurat and UMAP coordinates were generated using first 50 components returned by PCA. Cell types were determined in the Peng dataset using annotations provided by the authors and in the Schlesinger and Elyada datasets using panels of gene markers described in the results (Figure S1).

**Analysis of TCGA database.** Clinical data associated with the TCGA PAAD database was queried using the cBioPortal website ^10, 11^. First, misclassified and non-PDAC patient samples were excluded. Survival analysis was performed between groups representing the top and bottom 25% of samples by expression of a given gene. Statistics were computed using the log rank test.

1. Engle DD, Tiriac H, Rivera KD, et al. The glycan CA19-9 promotes pancreatitis and pancreatic cancer in mice. Science 2019;364:1156-1162.

2. Bankhead P, Loughrey MB, Fernandez JA, et al. QuPath: Open source software for digital pathology image analysis. Sci Rep 2017;7:16878.

3. Apaolaza PS, Petropoulou PI, Rodriguez-Calvo T. Whole-Slide Image Analysis of Human Pancreas Samples to Elucidate the Immunopathogenesis of Type 1 Diabetes Using the QuPath Software. Front Mol Biosci 2021;8:689799.

4. Maurer C, Holmstrom SR, He J, et al. Experimental microdissection enables functional harmonisation of pancreatic cancer subtypes. Gut 2019;68:1034-1043.

5. Maurer HC, Olive KP. Laser Capture Microdissection on Frozen Sections for Extraction of High-Quality Nucleic Acids. Methods Mol Biol 2019;1882:253-259.

6. Schlesinger Y, Yosefov-Levi O, Kolodkin-Gal D, et al. Single-cell transcriptomes of pancreatic preinvasive lesions and cancer reveal acinar metaplastic cells' heterogeneity. Nat Commun 2020;11:4516.

7. Elyada E, Bolisetty M, Laise P, et al. Cross-Species Single-Cell Analysis of Pancreatic Ductal Adenocarcinoma Reveals Antigen-Presenting Cancer-Associated Fibroblasts. Cancer Discov 2019;9:1102-1123.

8. Peng J, Sun BF, Chen CY, et al. Single-cell RNA-seq highlights intra-tumoral heterogeneity and malignant progression in pancreatic ductal adenocarcinoma. Cell Res 2019;29:725-738.

9. Stuart T, Butler A, Hoffman P, et al. Comprehensive Integration of Single-Cell Data. Cell 2019;177:1888-1902 e21.

10. Cerami E, Gao J, Dogrusoz U, et al. The cBio cancer genomics portal: an open platform for exploring multidimensional cancer genomics data. Cancer Discov 2012;2:401-4.

11. Gao J, Aksoy BA, Dogrusoz U, et al. Integrative analysis of complex cancer genomics and clinical profiles using the cBioPortal. Sci Signal 2013;6:pl1.
