# Supplementary material for: Eicosanoids in the Pancreatic Tumor Microenvironment—A Multicellular, Multifaceted Progression
Source: Gastro Hep Adv. 2022 Jun 11;1(4):682–97. doi: 10.1016/j.gastha.2022.02.007 (PMC9583893; doi:10.1016/j.gastha.2022.02.007)
Supplement: Table A1 [file mmc4.docx]

| **Antibody** | **Company** | **Catalog #** | **Dilution, IF** | **Dilution, IHC** |
| --- | --- | --- | --- | --- |
| Ac-α-tubulin | Sigma Aldrich | T7451 | 1:200 | N/A |
| αSMA | Santa Cruz | sc-32251 | 1:500 | N/A |
| Alox5 | Novus | NB110-58748 | 1:5000 | N/A |
| CD45 | Biolegend | 103102 | 1:100 | N/A |
| COX1 | Santa Cruz | sc-1754 | 1:100 | N/A |
| COX2 | Abcam | ab15191 | 1:500 | N/A |
| γActin | Santa Cruz | sc-65638 | 1:100 | N/A |
| PTGES | Novus | NBP1-87852 | 1:200 | 1:200 |
| PTGIS | Abcam | ab23663 | 1:1000 | 1:2000 |
| TBXAS1 | Sigma Aldrich | HPA031259 | 1:1000 | 1:2000 |

**Table S1. Primary antibodies used in immunofluorescence (IF) and immunohistochemistry (IHC) studies.**
