# Supplementary material for: Eicosanoids in the Pancreatic Tumor Microenvironment—A Multicellular, Multifaceted Progression
Source: Gastro Hep Adv. 2022 Jun 11;1(4):682–97. doi: 10.1016/j.gastha.2022.02.007 (PMC9583893; doi:10.1016/j.gastha.2022.02.007)
Supplement: Table A2 [file mmc5.docx]

| **Gene** | **Forward primer** | **Reverse primer** |
| --- | --- | --- |
| Alox5 | ACT ACA TCT ACC TCA GCC TCA TT | GGT GAC ATC GTA GGA GTC CAC |
| Hpgds  (exons 3-4) | TAC AAT CCA GAG CCT CG | ACA TTG CTC CAA CGC TGT CT​ |
| Ptgds | TGC AGC CCA ACT TTC AAC AAG | TGG TCT CAC ACT GGT TTT TCC T |
| Ptges1 | GGA TGC GCT GAA ACG TGG A | CAG GAA TGA GTA CAC GAA GCC |
| Ptges2 | CTT CCT TCG ACT ACA TTG TCC G | GGC GCT TGC TGA TGA GGT A |
| Ptges3 | TGT TTG CGA AAA GGA GAA TCC G | ACC CAT GTG ATC CAT CAT CTC A |
| Ptgs1 | ATG AGT CGA AGG AGT CTC TCG | GCA CGG ATA GTA ACA GGG A |
| Ptgs2 | TTC CAA TCC ATG TCA AAA CCG T | AGT CCG GGT ACA GTC ACA CTT |
| Rplp0 | GTG CTG ATG GGC AAG AAC | AGG TCC TCC TTG GTG AAC |

**Table S2. Primers used in qRT-PCR studies.**
