# Supplementary material for: Eicosanoids in the Pancreatic Tumor Microenvironment—A Multicellular, Multifaceted Progression
Source: Gastro Hep Adv. 2022 Jun 11;1(4):682–97. doi: 10.1016/j.gastha.2022.02.007 (PMC9583893; doi:10.1016/j.gastha.2022.02.007)
Supplement: Figure A1 [file mmc6.pdf]

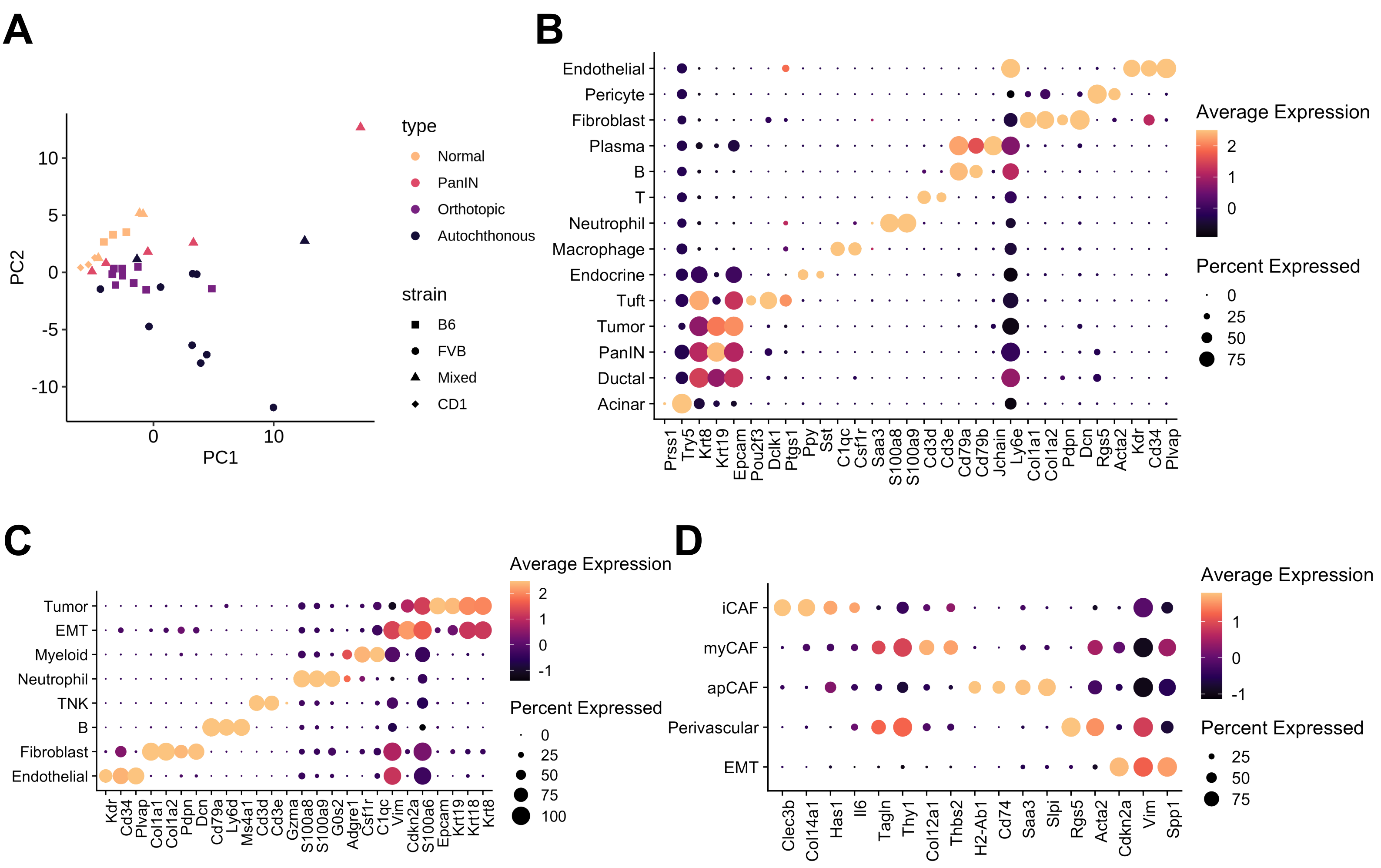

Figure S1. Eicosanoid signatures and cell type-specific markers in murine models of pancreatic tumorigenesis. (**A**) PCA plot comparing eicosanoid signatures of normal pancreata, PanIN-bearing pancreata, orthotopic PDAC tumors, or autochthonous PDAC tumors. (**B**) Dotplot of average and percent gene expression for cell type markers in a scRNA-seq dataset derived from Schlesinger et al. (**C**) Dotplot of average and percent gene expression for cell type markers in the entire unenriched dataset and (**D**) the fibroblast-enriched dataset generated from Elyada et al. EMT, epithelial to mesenchymal transition; TNK, T and natural killer cells; iCAF, inflammatory cancer-associated fibroblast; myCAF, myofibroblastic CAF; apCAF, antigen-presenting CAF.
