# Supplementary material for: Eicosanoids in the Pancreatic Tumor Microenvironment—A Multicellular, Multifaceted Progression
Source: Gastro Hep Adv. 2022 Jun 11;1(4):682–97. doi: 10.1016/j.gastha.2022.02.007 (PMC9583893; doi:10.1016/j.gastha.2022.02.007)
Supplement: Figure A2 [file mmc7.pdf]

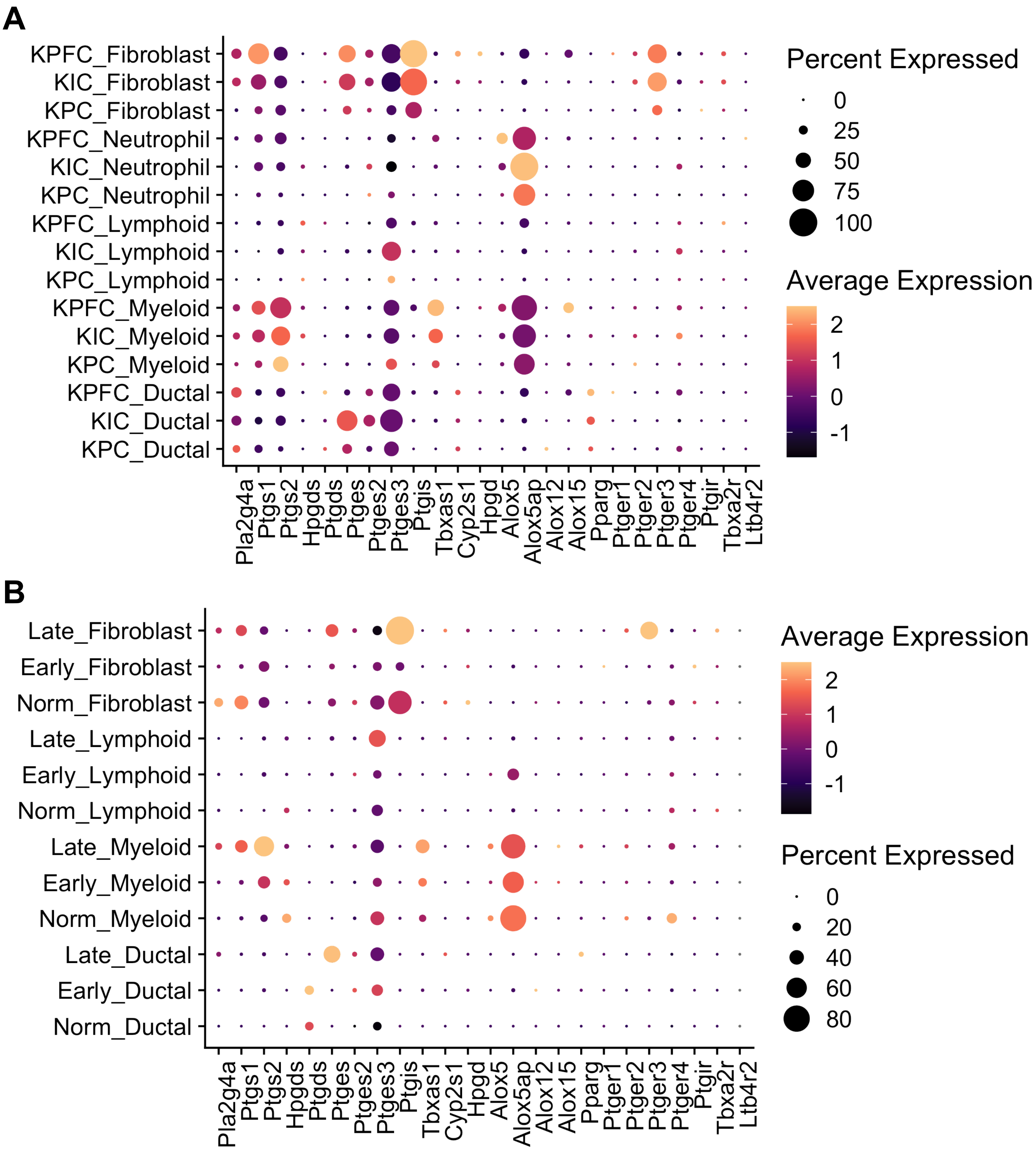

Figure S2. Eicosanoid pathway gene expression in multiple murine models of PDAC. Dotplots of eicosanoid synthase and receptor gene expression in normal pancreas and tumor epithelium organized by (**A**) mouse model or (**B**) stage of disease progression from the datasets described in Hosein et. al. KPC, LSL-KrasG12D;Trp53R172H;Ptf1aCre/+; KIC, LSL-KrasG12D; Ink4afl/fl;Ptf1aCre/+; KPFC, LSL-KrasG12D;Trp53fl/fl; Pdx1Cre/+. Norm, normal; Early, early lesions; Late, PDAC.
