# Supplementary material for: Eicosanoids in the Pancreatic Tumor Microenvironment—A Multicellular, Multifaceted Progression
Source: Gastro Hep Adv. 2022 Jun 11;1(4):682–97. doi: 10.1016/j.gastha.2022.02.007 (PMC9583893; doi:10.1016/j.gastha.2022.02.007)
Supplement: Figure A3 [file mmc8.pdf]

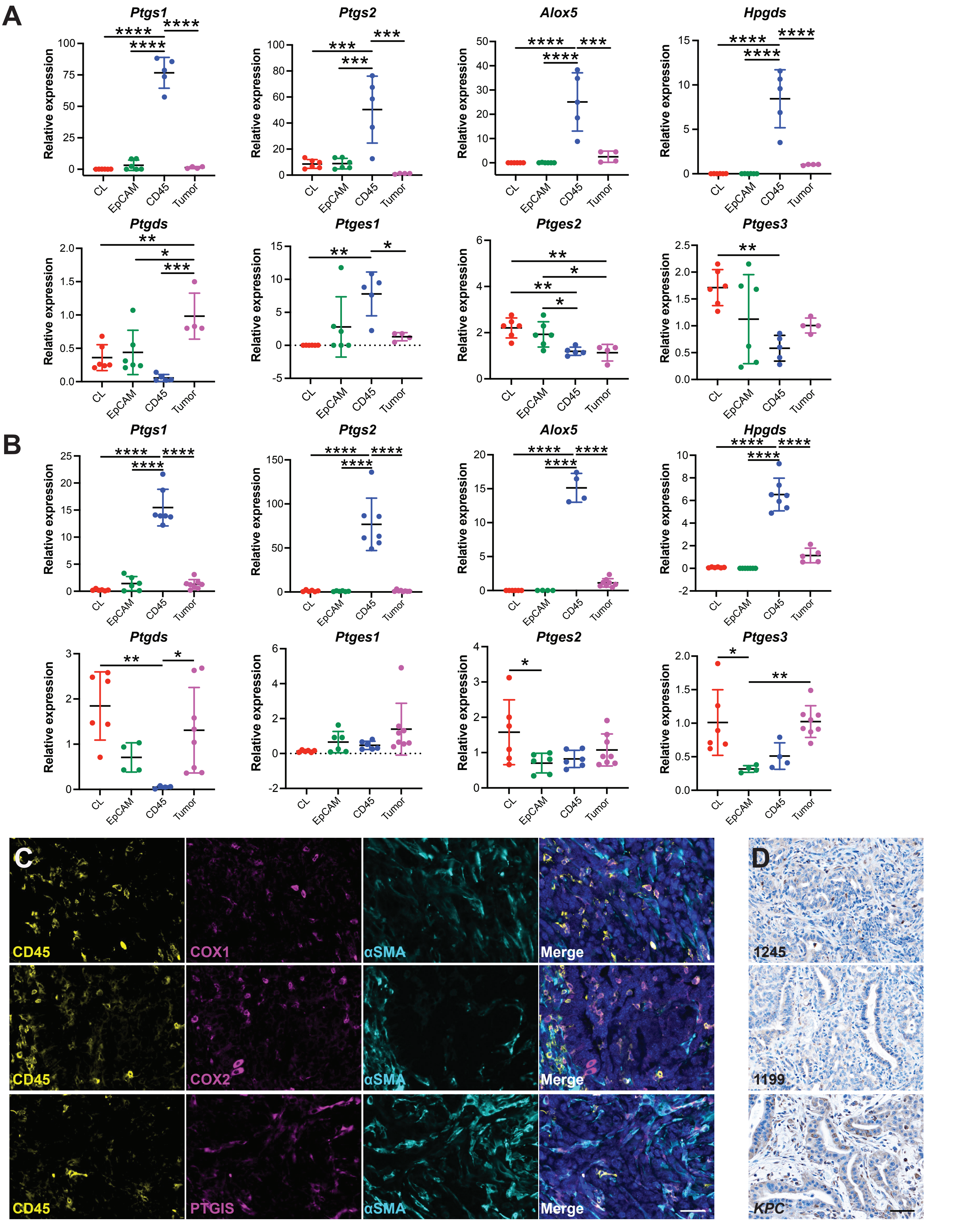

Figure S3. Cell type-specific expression of eicosanoid synthases in orthotopic models of PDAC. qRT-PCR for select eicosanoid synthases in (A) the FC-1245 or (B) the FC-1199 cell lines (CL), FACS collected EpCAM+ tumor or CD45+ immune cells, or whole orthotopic tumors. (C) Co-IF for COX1, COX2, or PTGIS (magenta) and CD45 (yellow), SMA (cyan), and DAPI (blue) in orthotopic tumors generated from the FC-1245 cell line. (D) IHC for COX2 in orthotopic tumors generated from FC-1245 or FC-1199 or a KPC tumor. Scale bars, 50 µm. *, $p < 0.05$; **, $p < 0.01$; ***, $p < 0.001$; ****, $p < 0.0001$.
