# Supplementary material for: Eicosanoids in the Pancreatic Tumor Microenvironment—A Multicellular, Multifaceted Progression
Source: Gastro Hep Adv. 2022 Jun 11;1(4):682–97. doi: 10.1016/j.gastha.2022.02.007 (PMC9583893; doi:10.1016/j.gastha.2022.02.007)
Supplement: Figure A4 [file mmc9.pdf]

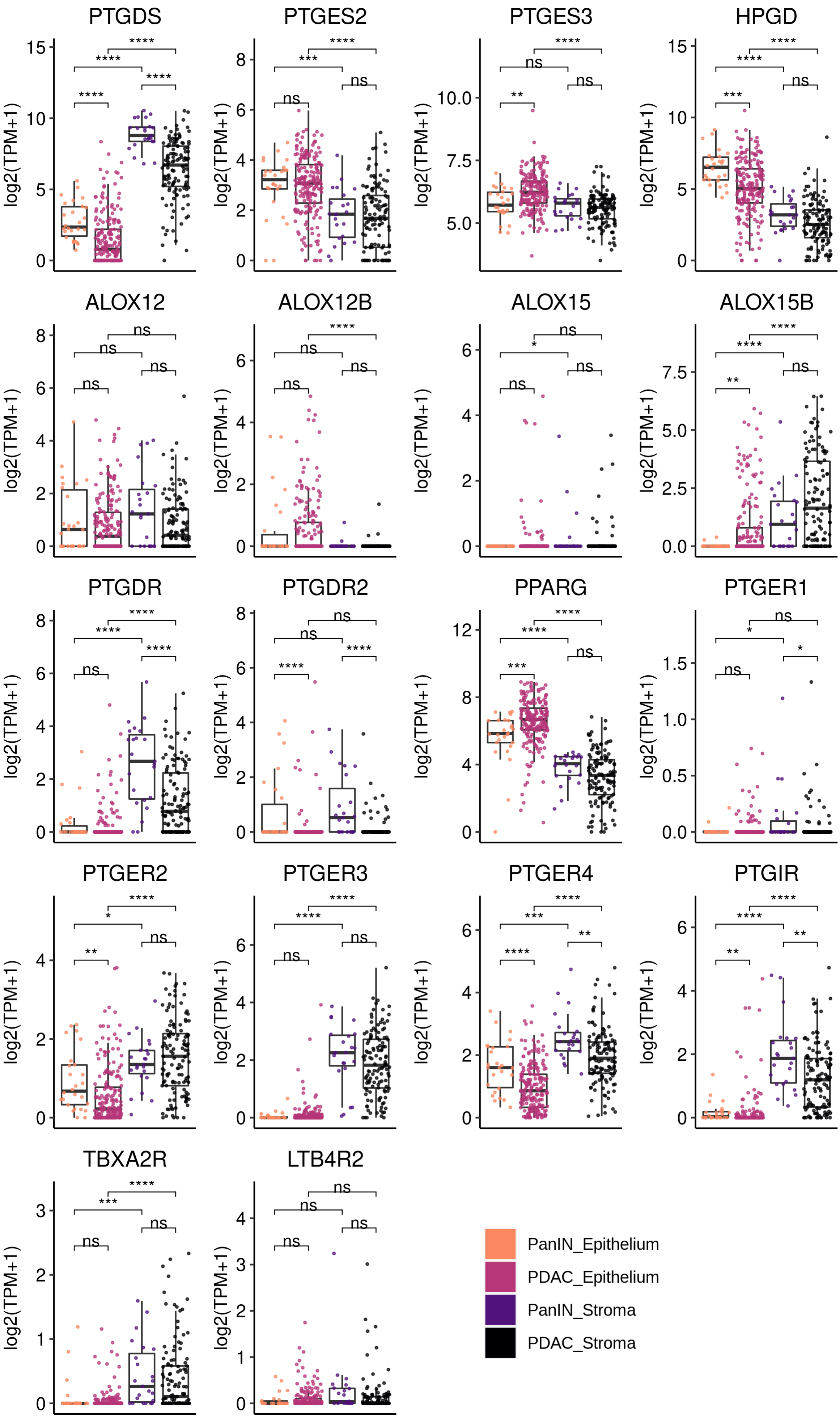

Figure S4. Eicosanoid pathway gene expression in the epithelium or stroma of human pre-invasive disease and PDAC. Boxplots comparing gene expression of eicosanoid synthases (log2(TPM+1)) in microdissected stroma and epithelium from PanIN or PDAC. *, $p < 0.05$; **, $p < 0.01$; ***, $p < 0.001$; ****, $p < 0.0001$.
