# Supplementary material for: Eicosanoids in the Pancreatic Tumor Microenvironment—A Multicellular, Multifaceted Progression
Source: Gastro Hep Adv. 2022 Jun 11;1(4):682–97. doi: 10.1016/j.gastha.2022.02.007 (PMC9583893; doi:10.1016/j.gastha.2022.02.007)
Supplement: Figure A5 [file mmc10.pdf]

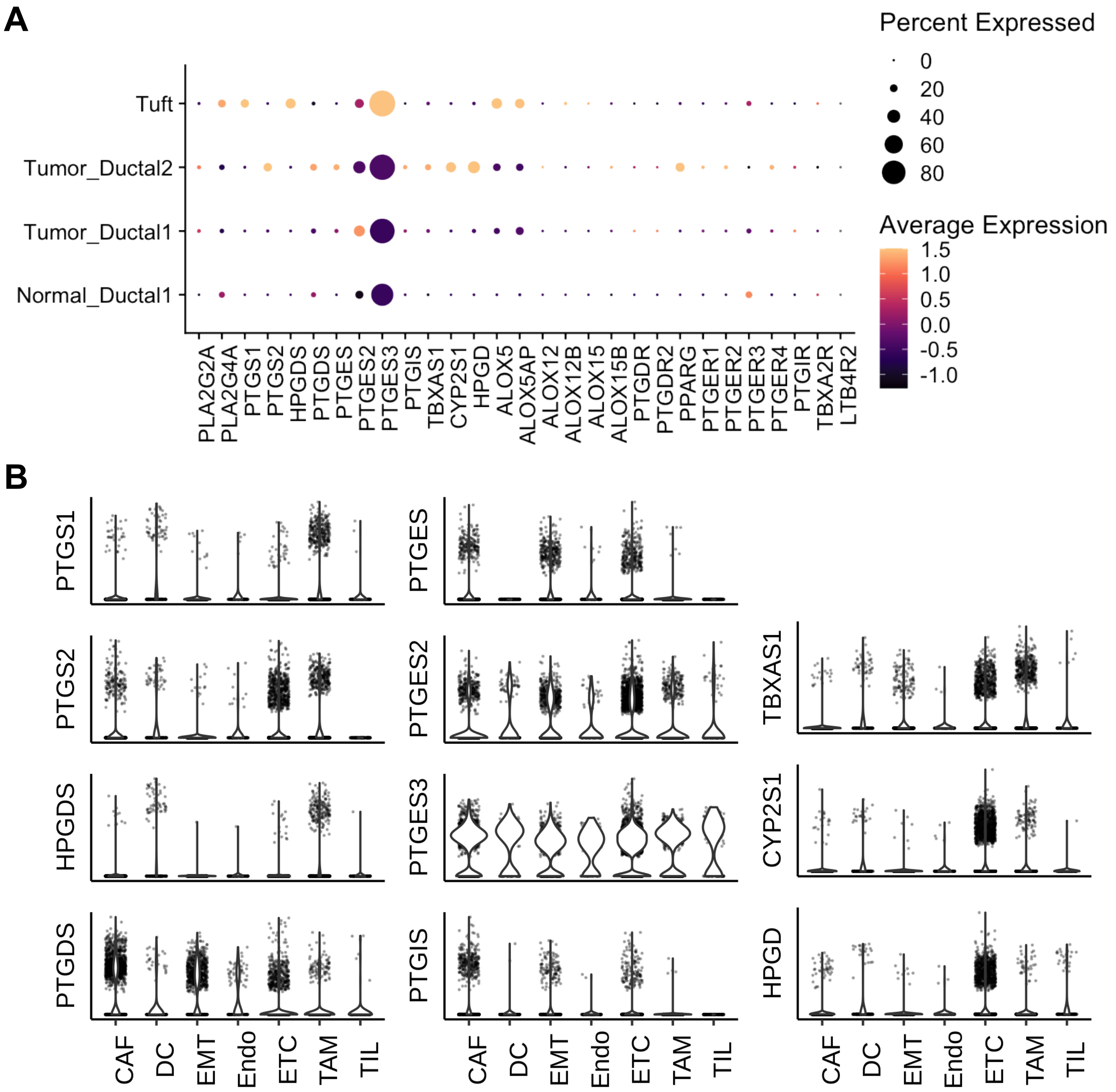

Figure S5. Eicosanoid synthase expression in human PDAC. (**A**) Heatmap of eicosanoid synthase and receptor gene expression in normal pancreas and tumor epithelium, from the dataset described in Peng et al. (B) Violin plots of eicosanoid synthase expression from a human PDAC scRNA-seq dataset described in Lin et al. CAF, cancer-associate fibroblast; DC, dendritic cells; EMT, epithelial to mesenchymal transition; Endo, endothelial; ETC, epithelial tumor cells; TAM, tumor-associated macrophages; TIL, tumor-associated lymphocytes.
