# Supplementary material for: Eicosanoids in the Pancreatic Tumor Microenvironment—A Multicellular, Multifaceted Progression
Source: Gastro Hep Adv. 2022 Jun 11;1(4):682–97. doi: 10.1016/j.gastha.2022.02.007 (PMC9583893; doi:10.1016/j.gastha.2022.02.007)
Supplement: Figure A6 [file mmc11.pdf]

A

Significant correlations in IHC analyses	R	P-value
PTGES normal ductal epithelium vs. PTGES normal ductal stroma	0.629**	0.003x10^{-5}
PTGES normal ductal epithelium vs. TBXAS1 normal ductal epithelium	0.506**	0.002x10^{-2}
PTGES normal ductal stroma vs. TBXAS1 normal ductal epithelium	0.433**	0.003x10^{-1}
PTGES normal ductal stroma vs. TBXSAS1 normal ductal stroma	0.399**	0.001
PTGES Metaplasia epithelium vs. TBXAS1 Metaplasia epithelium	0.258*	0.035
PTGES HG PanIN epithelium vs. PTGES HG PanIN stroma	0.488*	0.025
PTGES HG PanIN epithelium vs. TBXAS1 HG PanIN epithelium	0.548*	0.010
PTGIS normal ductal stroma vs. PTGIS normal ductal epithelium	-0.347**	0.005
PTGIS normal ductal epithelium vs. TBXAS1 normal ductal epithelium	-0.356**	0.004
PTGIS normal ductal stroma vs. PTGES normal ductal epithelium	0.468**	0.009x10^{-2}
PTGIS normal ductal stroma vs. PTGES normal ductal stroma	0.427**	0.004x10^{-1}
PTGIS normal ducal stroma vs. TBXAS1 normal ductal epithelium	0.470**	0.009x10^{-2}
PTGIS normal ductal stroma vs. TBXAS1 normal ductal stroma	0.360**	0.003
PTGIS Metaplasia stroma vs. TBXAS1 Metaplasia stroma	0.406**	0.001
PTGIS WD PDAC stroma vs. TBXAS1 WD PDAC stroma	0.387**	0.005x10^{-1}
PTGIS PD PDAC stroma vs. PTGES PD PDAC stroma	-0.313*	0.032
TBXAS1 normal ductal epithelium vs. TBXAS1 normal ductal stroma	0.458**	0.001x10^{-1}

B

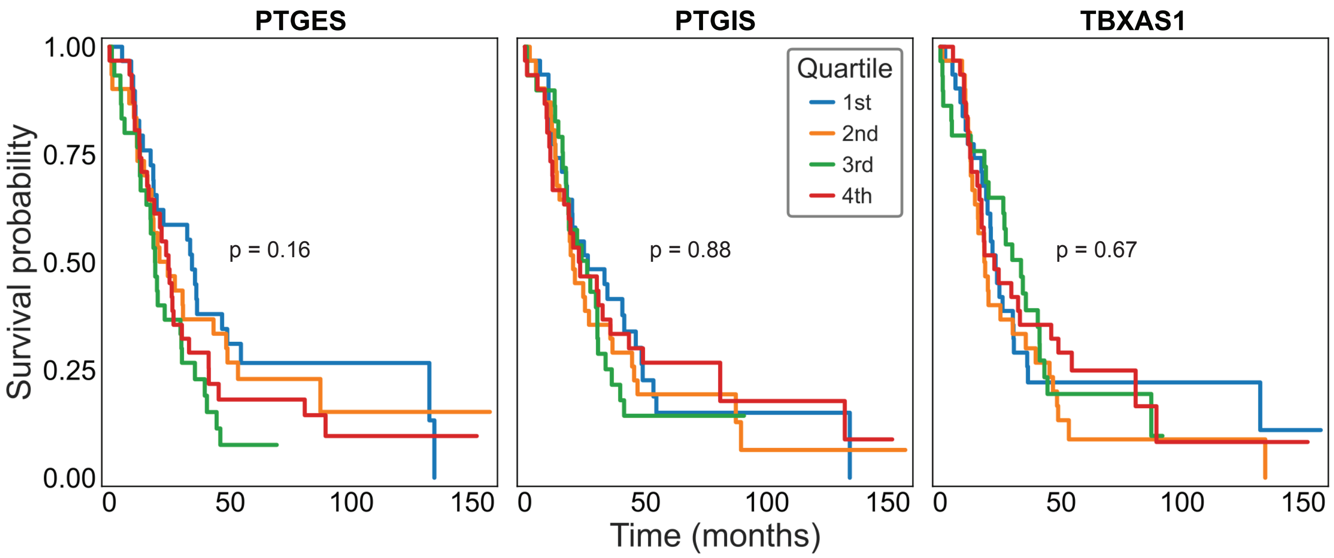

Figure S6. Immunohistochemical analysis of eicosanoid sythases in PDAC samples and patient survival associated with expression of select eicosanoid pathway genes. (**A**) Table of significant correlations identified in the IHC analysis of PTGES, PTGIS, and TBXAS1 expression. Green, normal tissue; purple, diseased tissue. HG, high grade; WD, well differentiated; PD, poorly differentiated. (**B**) Survival curves for stroma-specific expression of PTGES, PTGIS, or TBXAS1 generated from the Maurer et al., dataset representing 124 PDAC patients.
